# Activation of Insulin Signaling in Adipocytes and Myotubes by *Sarcopoterium Spinosum* Extract

**DOI:** 10.3390/nu11061396

**Published:** 2019-06-21

**Authors:** Michaella Ben-Shachar, Konstantin Rozenberg, Nir Skalka, Ayala Wollman, Michal Michlin, Tovit Rosenzweig

**Affiliations:** Departments of Molecular Biology and Nutrition Sciences, Ariel University, Ariel 40700, Israel; 0584409512bs@gmail.com (M.B.-S.) rozenberg.kos@gmail.com (K.R.); nirskalka@gmail.com (N.S.); ayalalumer22@gmail.com (A.W.); michal.michlin@gmail.com (M.M.)

**Keywords:** diabetes, glucose transport, insulin action, insulin signaling

## Abstract

*Sarcopoterium spinosum* (*S. spinosum*) is a medicinal plant, traditionally used as an antidiabetic remedy. Previous studies demonstrated its beneficial properties in the treatment of insulin resistance. The aim of this study was to further clarify the effect of *S. spinosum* extract (SSE) on insulin signaling. Phosphoproteomic analysis, performed in 3T3-L1 adipocytes treated with SSE, revealed the activation of insulin receptor pathways. SSE increased Glut4-facilitated glucose uptake in adipocytes, with an additive effect between SSE and insulin. While the maximal effect of insulin on glucose uptake was found at days 15–16 of differentiation, SSE-induced glucose uptake was found at an earlier stage of differentiation. Inhibition of PI3K and Akt blocked SSE-dependent glucose uptake. Western blot analysis, performed on 3T3-L1 adipocytes and L6 myotubes, showed that in contrast to insulin action, Akt was only marginally phosphorylated by SSE. Furthermore, GSK3β and PRAS40 phosphorylation as well as glucose uptake were increased by the extract. SSE also induced the phosphorylation of ERK similar to insulin. In conclusion, SSE activates insulin signaling, although the upstream event mediating its effects should be further clarified. Identifying the active molecules in SSE may lead to the development of new agents for the treatment of insulin resistance.

## 1. Introduction

Although the number of approved anti-diabetic medications is growing, the goal of treatment, which is maintaining a HbA1C (hemoglobin A1C) of ≤7% remains difficult to achieve [1,2]. Type 2 diabetes mellitus (T2DM) is a disease characterized by an impaired insulin action, and is considered one of the major causes for illness and premature death, which results from severe complications of the disease [3,4]. These startling statistics illustrate the inability of current anti-diabetes drugs to retard disease progression and the continued need for further research and development of alternative drugs with novel mechanisms to slow disease progression and complications.

Normally, insulin binding to its receptor induces auto-phosphorylation of the receptor, leading to a subsequent recruitment of docking proteins and phosphorylation of various downstream substrates. This cascade leads to the activation of at least two parallel pathways: the PI3K-Akt and the MAPK pathway. The metabolic functions of insulin, i.e., increasing glucose uptake and glycogen synthesis and inhibiting gluconeogenesis and lipolysis, are regulated by the PI3K-Akt pathway. Once activated, PI3K increases the levels of phosphatidyl-inositol (3,4,5)-triphosphate (PIP3), enabling the binding of Akt and PDK1, both possessing PH (pleckstrin homology) domains, to the membrane [5,6,7,8]. This co-localization enables phosphorylation of Akt by PDK1 at the catalytic domain leading to Akt activation. An additional phosphorylation on S473, promoted by mTOR, further increases Akt activity and affects substrate recognition. Once activated, Akt is translocated to various subcellular compartments—including the nucleus—where it phosphorylates several substrates, including GSK3β, PRAS40, AS160, FOXO proteins and others [9,10,11]. This enables signal divergence to the various metabolic pathways regulated by insulin Including GLUT4 translocation, glucose uptake [12], glycogen and protein synthesis [13]. A disruption in the transmission of insulin signaling, recognized as insulin resistance, is the main pathological process leading to T2DM, and is also an underlying pathology of other disorders included under the spectrum of “metabolic syndrome”. IRS1/IRS2 downregulation following hyperinsulinemia, metabolic-related inflammation, and over-nutrition, along with the resulting Akt inactivation and Foxo1 activation are involved in the progression of metabolic syndrome in humans [14]. A defective insulin action, reduction in insulin-stimulated Akt phosphorylation on ser473/4 and thr308/9, and reduction in the activity of all Akt isoforms were demonstrated in patients with T2DM [15,16]. The reduced response to insulin causes pancreatic β-cells to increase insulin secretion, leading to a state of chronic hyperinsulinemia, which was found to be a result and driver of insulin resistance [17]. This scenario leads to a progressive worsening and perpetuation of the disease. In parallel, dysfunction of β-cells that cannot compensate adequately for insulin resistance promotes the outbreak of the disease [18]. Accordingly, the search for new enhancers of glucose transport is of high importance.

The plant kingdom may be viewed as a huge bank of compounds with different biological activities, which can be used for treating various diseases, either when consumed as dietary supplements or as a basis for the development of botanically or chemically purified drugs. For instance, metformin, the first-line medication for T2DM, was developed on the basis of guanidine isolated from *Gallega officinalis* [19]. Phlorizin, the first SGLT2 (sodium/glucose transporter 2) inhibitor, is a naturally occurring polyphenol found in some plants, including the bark of apple trees [20]. Although more than 400 plants have been suggested by traditional medicine for the treatment of diabetes [21,22], only a few have been thoroughly evaluated. Among the medicinal plants used against T2DM in folklore medicine is *Sarcopoterium spinosum* (*S. spinosum)*, the focus of this research.

*Sarcopoterium spinosum* L. (Rosaceae), also known as the thorny burnet, is mentioned as a medicinal plant in most ethno-pharmacological surveys performed in Israel and Jordan, and is often used in traditional Arab and Bedouin medicine [23,24]. Its primary traditional use is of the aqueous extract of the plant, prepared from its root bark, for the treatment of T2DM [24,25,26,27,28]. Indeed, several studies have investigated and established the anti-diabetic function of *S. spinosum*. In vitro experiments performed in our laboratory demonstrated that *S. spinosum* extract (SSE) exerts insulin-like effects, including increased glucose uptake by skeletal muscle cells, adipocytes, and hepatocytes; increased GSK3β phosphorylation in myotubes; and reduced lipolysis in adipocytes [29]. In vivo studies further support the anti-diabetic properties of the herb, which reduced blood glucose levels in both normal rabbits and in alloxan-treated rats [30]. We further validated this finding [29] using the KK-Ay mice, which are a spontaneously (genetically) developing diabetes model characterized by hyperphagia, obesity, severe insulin resistance, and hyperglycemia. All these disturbances were improved by the consumption of SSE, indicating that the most prominent mechanisms of action are those affecting the target tissues of insulin, mediated by improved insulin sensitivity or by mimicking insulin action, rather than by increasing insulin secretion [29,31]. We also demonstrated these beneficial properties of SSE in glucose-intolerant mice, induced by the consumption of a high-fat diet [32].

The mechanisms mediating the effect of *S. spinosum* on insulin sensitivity are currently unknown. We previously found that while *S. spinosum* induction failed to induce Akt phosphorylation on ser473, which is known to be an important signaling event required for GLUT4 translocation and glucose transport [33], this kinase was found to be translocated to the membrane and nucleus. The aim of this study was to further clarify the role of insulin signaling cascade in SSE action and the mechanisms mediating the stimulatory effect of SSE on glucose uptake.

## 2. Materials and Methods

### 2.1. S. Spinosum Extract Preparation

*Sarcopoterium spinosum* (L.) Spach. (Thorny burnet, local name: Natesh, Billan (Arabic), Sira Kotzanit (Hebrew)) was collected from the wild in the area around Ariel University. A voucher specimen of the plant was deposited in the Israel National Herbarium at the Hebrew University of Jerusalem (No. HUJ 102531). *S. spinosum* aqueous root extract was prepared, as described previously, by boiling 100 g roots/L [29,31]. The extract was lyophilized and kept at −20 °C, giving a yield of 0.7% dry material. The dried extract was dissolved again in double-distilled water (DDW), according to the experimental requirements. Uniformity of the extract was ensured as described previously [32,34].

### 2.2. Cell Culture

3T3-L1 pre-adipocytes (ATCC, passage number < 15) were cultured and induced to differentiate as described before [31]. L6 myoblasts (ATCC, passage number < 25) were grown in MEM-α containing 25 mM glucose, 10% FCS, 2 mM glutamine, and 1% ampicillin. Experiments were performed on differentiated myotubes. L6 differentiation was induced as described in our previous studies [31].

### 2.3. Sample Preparation and Phosphopeptide Enrichment for Mass Spectrometry and Phosphopeptide Quantitation

3T3-L1 adipocytes (14th day of differentiation) were treated for 30 min by either insulin (100 nM), *S. spinosum* extract (70 μg/mL), or vehicle. The treatment dose and time of exposure were chosen according to our previous studies [31,34]. Samples were prepared and enriched using the method reported by Ruprecht et al. [35]. Briefly, cell pellets were lysed with urea and digestion with trypsin. Phosphopeptides were enriched from the total protein digest using Immobilized Metal Affinity Chromatography as described [35].

### 2.4. Liquid Chromatography and Mass Spectrometry

Each sample was loaded using split-less nano-Ultra Performance Liquid Chromatography (10 kpsi nanoAcquity; Waters, Milford, MA, USA), coupled to a quadrupole orbitrap mass spectrometer (Q Exactive Plus, Thermo Scientific, San Jose, CA, USA). Data was acquired in a data-dependent acquisition mode, using a Top20 method as described [36].

### 2.5. Data Processing and Analysis

Raw data was imported into the Expressionist^®^ software version 10.5 (Genedata) and processed as described here. The software was used for retention time alignment and peak detection of precursor peptides. A master peak list was generated from all MS/MS events and sent for database searching using Mascot v2.5.1 (Matrix Sciences). Data were searched against the mouse sequences UniprotKB (http://www.uniprot.org/) appended with common laboratory contaminant proteins. Fixed modification was set to carbamidomethylation of cysteines and variable modifications were set to oxidation of methionines, phosphorylation of S, T, or Y and deamidation of N or Q. Search results were then filtered using the PeptideProphet algorithm to achieve a maximum false discovery rate of 1% at the protein level. Peptide identifications were imported back to Expressionist to annotate identified peaks. Quantification of proteins from the peptide data was performed using an in-house script. Data were normalized based on the total ion current. Protein abundance was obtained by summing the three most intense, unique peptides per protein. A Student’s *t*-Test, after logarithmic transformation, was used to identify significant differences across the biological replica. Fold changes were calculated based on the ratio of arithmetic means of the case versus control samples.

### 2.6. Bioinformatics

All quantified phosphopeptides were analyzed using Ingenuity Pathway Analysis (IPA, Ingenuity Systems, Redwood City, CA, USA) by core analysis. The canonical pathways overrepresented by insulin or SSE were generated based on the Ingenuity Pathways Knowledge Base.

### 2.7. Glucose Uptake

Cells were preincubated for 2 h in serum-free DMEM. Starvation media was replaced, and cells were treated for 30 min with SSE (70 µg/mL) or 100 nM insulin, in the presence or absence of selective inhibitors (wortmannin, Akt inhibitor VIII, GSK690693, PD98059 and SB2035810, all were purchased from Calbiochem). Cells were washed twice with phosphate buffer saline (PBS), followed by 5 min incubation in 37 °C in PBS solution containing 0.1 mM 2-deoxy glucose (2DG) and 0.5 μCi [^3^H]-2DG. Cytochalasin-B (20 µM) was used for the measurement of non-specific glucose uptake. Cells were then washed 3 times with cold PBS and incubated in 1% SDS in order to lyse the cells. The contents of each well were transferred to a different plate containing Optiphase scintillation liquid and counted using a MicroBeta counter (Perkin-Elmer).

### 2.8. Western Blot Analysis

Protein lysates of differentiated L6 and 3T3-L1 cells were prepared using RIPA buffer supplemented with protease and phosphatase inhibitors. The samples were homogenized and centrifuged at 14,000 rpm for 20 min. The supernatant was collected, and protein concentration was measured using the Bradford method. Protein was separated (20 μg per lane) by SDS-polyacrylamide gel electrophoresis. Proteins were electrophoretically transferred onto nitrocellulose membranes. The membranes were blocked in 5% dry milk, incubated with the appropriate antibody solutions (5% BSA in 0.01% TBST) and proteins were immunodetected using the enhanced chemiluminescence method. Primary antibodies were purchased from Cell Signalling Technologies, except anti-actin (MP Biomedicals). Secondary antibodies were purchased from Jackson Immuno Research.

### 2.9. Statistical Analysis

Values are presented as mean ± SEM. Statistical differences between the treatments and controls were tested by unpaired two-tailed Student’s *t*-test or one-way analysis of variance (ANOVA), followed by Bonferroni’s post-hoc testing when appropriate. Analysis was performed using the GraphPad Prism 5.0 software. A difference of *p* = 0.05 or less in the mean values was considered statistically significant.

## 3. Results

In order to support our previous studies demonstrating the anti-diabetic properties of SSE, and to further clarify its mechanism of action, a hypothesis-free, high throughput approach for global quantification of the phospho-proteome induced by *S. spinosum* was employed. A quantitative, label-free MS-based approach was performed, enabling the detection of serine/threonine phosphorylation of 5153 phospho-peptides.

We identified three groups of proteins: phosphopeptides regulated specifically by insulin, by SSE, or by both agents. (See supplementary Tables S1–S3 for lists of phosphopeptides, which were regulated by both SSE and insulin (Supplementary Table S1), only by SSE (Supplementary Table S2) and only by insulin (Supplementary Table S3)). The proteomics data was integrated with a network of protein–protein interactions and biological pathways to obtain a systems level view of phospho-proteome changes that occur following insulin and SSE treatment.

The phosphoserine/threonine dataset was filtered for peptides that were modulated upon insulin treatment. This generated a list of 545 phosphopeptides, corresponding to 322 proteins; most of these showed an increase in phosphorylation and only three of which displayed a decrease. The data were similarly filtered for peptides that were modulated upon SSE treatment, generating a list of 253 affected phosphopeptides, corresponding to 184 proteins; most of these showed an increase in phosphorylation and only six of which displayed a decrease.

A bioinformatics analysis (IPA, ingenuity pathway analysis) revealed that the top canonical signaling cascade with the highest likelihood to be activated by insulin (*p* = 1.28 × 10^−5^) and *S. spinosum* (*p* = 4.54 × 10^−7^) is the insulin signaling cascade (Table 1 and Table 2). Although both treatments activated this pathway, some phosphorylation was regulated by insulin and was not affected by *S. spinosum*, and vice versa.

**Figure 1:**
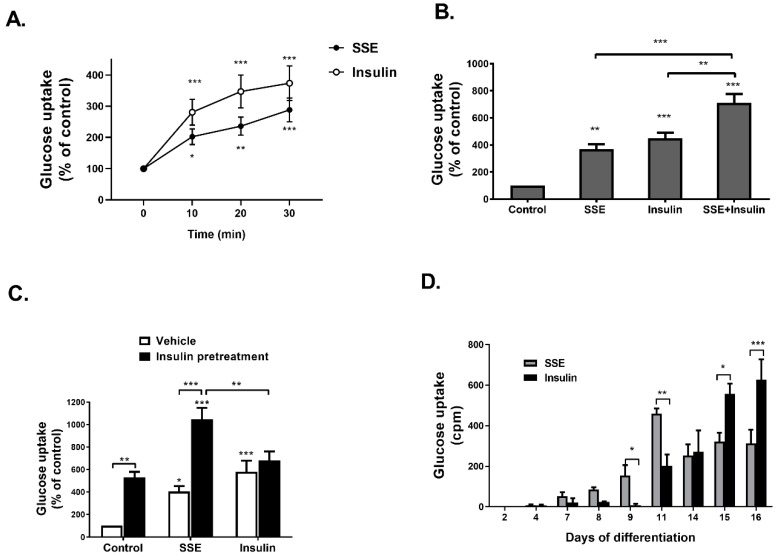
SSE- and insulin-induced glucose uptake in the same time course, with an additive effect. A-C were performed on 3T3-L1 adipocytes at the 14th day of differentiation. (**A**) 3T3-L1 adipocytes were treated with SSE or insulin for the indicated time before measuring glucose uptake. (**B**) 3T3-L1 adipocytes were treated with SSE, insulin or both, for 30 min before measuring glucose uptake. (**C**) 3T3-L1 adipocytes were treated with insulin or vehicle 24 h before treating the cells with either SSE or insulin for additional 30 min before measuring glucose uptake. (**D**) 3T3-L1 at different days of differentiation were treated with SSE or insulin for 30 min before measuring glucose uptake. In all experiments (**A**–**D**), the uptake of [3H]2-Deoxy-D-glucose into cells was determined as described in Material and Methods. The data represent the mean ± SEM of measurement made on six replicates in each of at least five independent experiments. * *p* < 0.05, ** *p* < 0.005, and *** *p* < 0.0005 compared to untreated cells by Student’s *t*-test (**A**) or analyzed by one-way ANOVA, followed by Bonferroni’s post-hoc testing (**B**–**D**).

SSE-induced glucose uptake by differentiated 3T3-L1 adipocytes with a similar time-course to insulin effect (Figure 1A). When cells were treated with both insulin and SSE, glucose uptake was enhanced in an additive manner (Figure 1B). In addition, in order to further demonstrate the additive effect of SSE over insulin, 3T3-L1 adipocytes were treated with insulin for 24 h before additional stimulation with either insulin or SSE (Figure 1C). Glucose uptake was significantly elevated by SSE over the effect of chronic insulin stimulation, while acute insulin stimulation failed to further enhance the effect of chronic insulin. In order to further support these observations, suggesting the utilization of different mechanisms of action by SSE for the activation of an insulin signaling cascade other than insulin, we followed glucose uptake by differentiating adipocytes at different stages of the process (Figure 1D).

While the efficiency of glucose transport was increased by insulin as the differentiation proceeded, achieving maximal rate of uptake at 15th–16th days of differentiation, SSE had different kinetics; it induced glucose uptake at earlier stages of differentiation (9th day), a maximal effect found on the 11th day, followed by a lower, stable rate of glucose transport during the 14th–16th days of differentiation.

**Figure 2:**
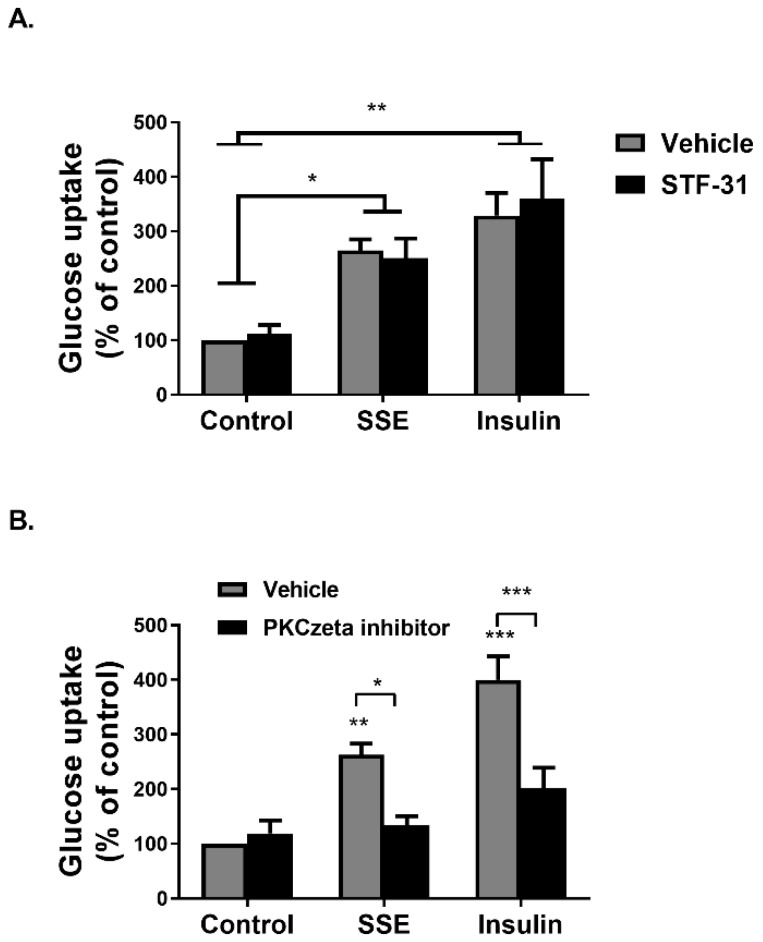
Induction of glucose uptake by SSE is facilitated by Glut4 transporters. (**A**,**B**) Differentiated 3T3-L1 adipocytes were treated with 5µM STF31 for 1 h (**A**) or 50 µM PKCzeta inhibitor (**B**) for 30 min before treatment with SSE or insulin for additional 30 min. Deoxy-D-glucose in cells was determined as described in Materials and Methods. Data are expressed as percent of basal uptake in control cells. The data represent the mean ± SEM of measurement made on six replicates in each of at least five independent experiments. * *p* < 0.05, ** *p* < 0.005, and *** *p* < 0.0005, analyzed by one-way ANOVA, followed by Bonferroni’s post-hoc testing.

Glucose transport into adipocytes and myotubes is facilitated either by the Glut1 transporter, considered to enhance basal glucose uptake, and Glut4, which is the transporter mediating insulin-dependent glucose uptake. Glut1 inhibition, using STF31, a selective inhibitor of this transporter [37], failed to inhibit SSE and insulin-dependent glucose uptake (Figure 2A). On the other hand, inhibition of PKCzeta, known to be a key player in the translocation of Glut4 to the plasma membrane [38], using a cell-permeable pseudosubstrate, blocked the effects of both insulin and SSE on glucose transport (Figure 2B), suggesting that in similarity to insulin, SSE utilizes Glut4 for the facilitation of glucose transport.

**Figure 3:**
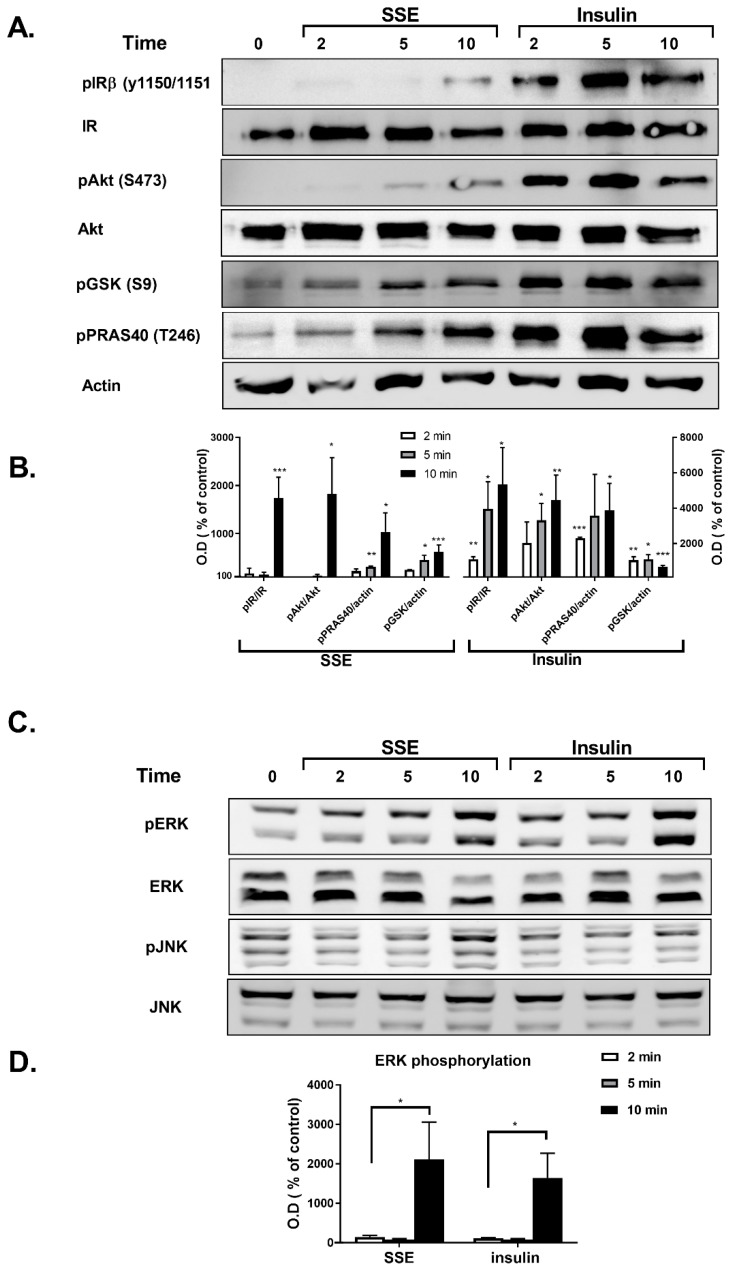
SSE phosphorylates components of the PI3K-Akt and MAPK pathway in L6 myotubes. L6 myotubes were treated with insulin or SSE for the indicated time. (**A**,**B**). Western blot analysis of proteins involved in PI3K-Akt pathway and a graph of optical densitometry (OD) measurements. (**C**) and (**D**). Western blot analysis of proteins involved in MAPK pathway and a graph of OD measurements. These are representative results of 3 independent experiments. Phosphorylation level was normalized to the expression of the proteins. Results are presented as % of control. Each bar represents the mean ± SE of data obtained in three separate experiments. * *p* < 0.05, ** *p* < 0.005, and *** *p* < 0.0005 vs. control, analyzed by Student’s *t*-test.

**Figure 4:**
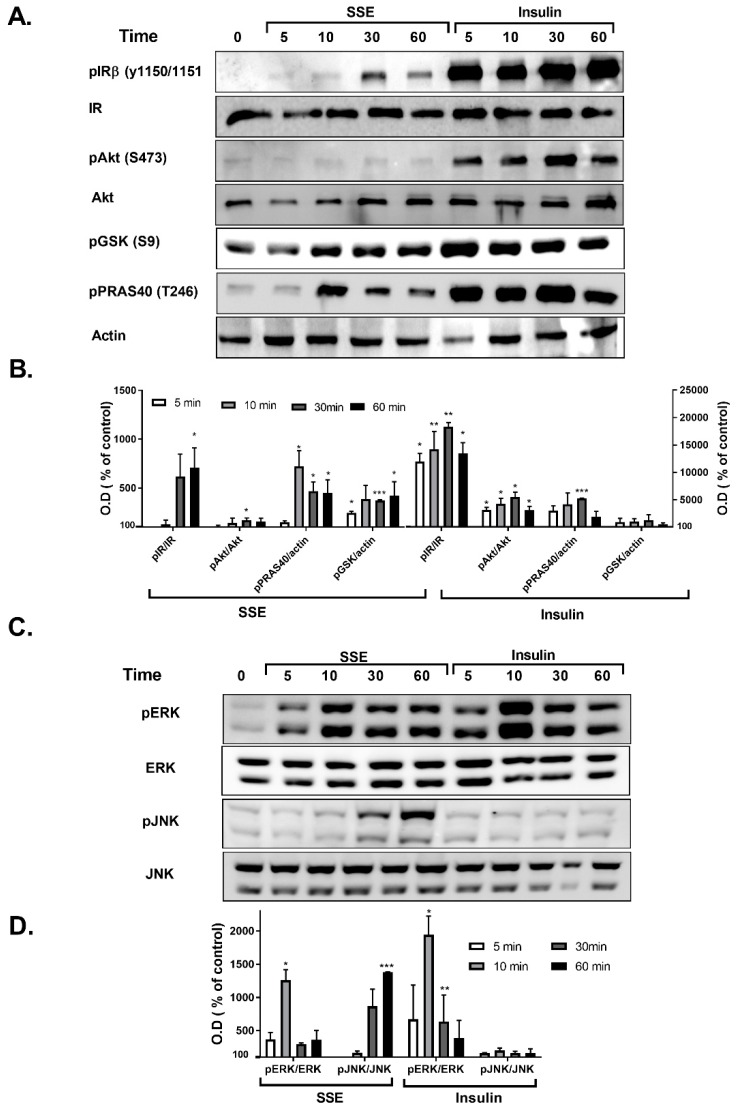
SSE phosphorylates components of the PI3K-Akt and MAPK pathway in 3T3-L1 adipocytes. 3T3-L1 adipocytes were treated with insulin or SSE for the indicated time. (**A**,**B**). Western blot analysis of proteins involved in PI3K-Akt pathway and a graph of optical densitometry (OD) measurements. (**C**,**D**). Western blot analysis of proteins involved in MAPK pathway and a graph of OD measurements. Phosphorylation level was normalized to the expression of the proteins. Results are presented as % of control. Each bar represents the mean ± SE of data obtained in three separate experiments. * *p* < 0.05, ** *p* < 0.005, and *** *p* < 0.0005 vs. control, analyzed by Student’s *t*-test.

Phosphorylation of several proteins, considered to play a key role in the transmission of the insulin signaling cascade, was measured in L6 myotubes and 3T3-L1 adipocytes treated by either insulin or SSE. Insulin binding to its receptor activates two major signaling cascades: the PI3K-Akt pathway and the MAPK pathway. We first followed the effect of SSE on the PI3K-Akt pathway, which is considered to mediate the metabolic effects of insulin. Insulin-induced phosphorylation of insulin receptor (IR), Akt, GSK3β and PRAS40 was rapidly increased following 2 and 5 min of treatment in L6 myotubes (Figure 3A,B) and 3T3-L1 adipocytes (Figure 4A,B), respectively. Unlike insulin, SSE had a very low stimulatory effect on IR phosphorylation in both cell lines, which was somewhat increased during the late stage of induction (10 min in L6 myotubes, 30–60 min in 3T3-L1 adipocytes). Similarly, Akt phosphorylation on ser473, a phosphorylation site considered as a biomarker for Akt activation, was almost undetected in SSE-treated cells. On the other hand, GSK3β and PRAS40 were phosphorylated earlier, following 5 or 10 min of SSE-treatment in L6 myotubes (Figure 3A,B) and 3T3-L1 adipocytes (Figure 4A,B), respectively.

The effect of SSE on MAPK pathway was also followed in L6 and 3T3-L1 adipocytes. Among the three different MAPK proteins; p38, JNK and ERK1/2, p38 were not phosphorylated by either insulin or SSE (data not shown). As expected [39], insulin increased the phosphorylation of ERK1/2, and did not affect JNK phosphorylation (Figure 3C,D and Figure 4C,D). Insulin and SSE induced the phosphorylation of ERK in the same time course. This phosphorylation was detected in both L6 and 3T3-L1, but was much higher in the latter cell line (Figure 3C,D and Figure 4C,D). Interestingly, SSE also induced a minor phosphorylation of JNK that was found in a different time course than ERK phosphorylation. This was detected in 3T3-L1 adipocytes, but not in L6 myotubes.

**Figure 5:**
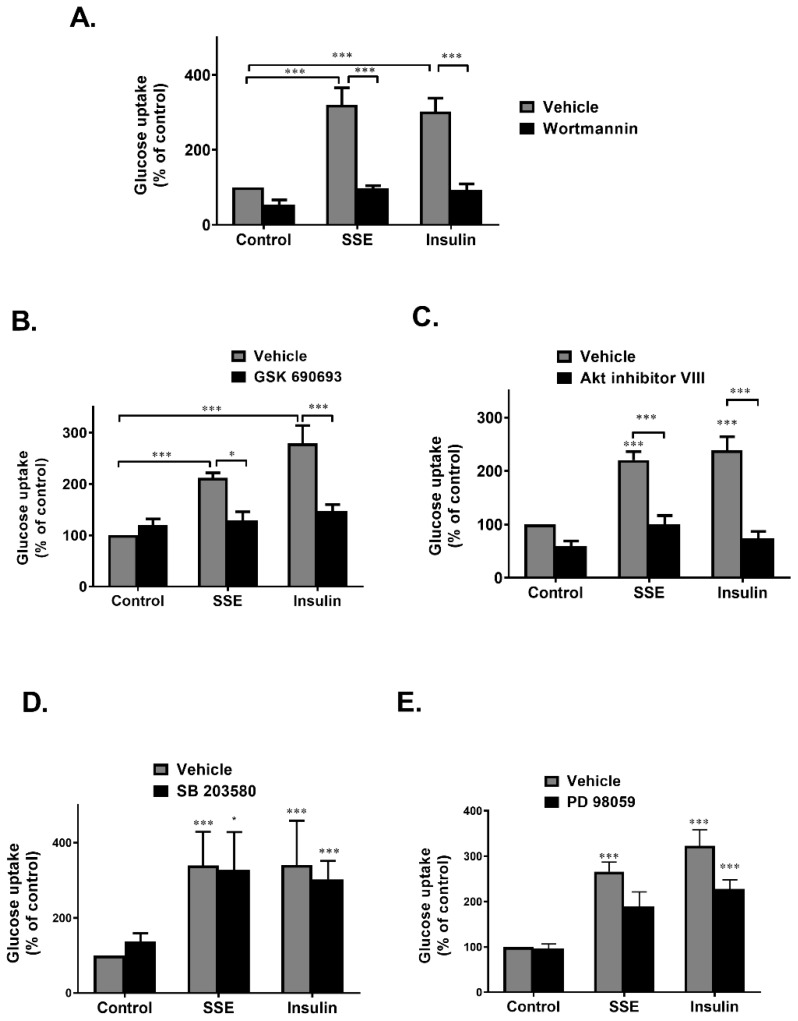
PI3K-Akt pathway is required to activate SSE-dependent glucose uptake. Differentiated 3T3-L1 adipocytes were treated with SSE (70 µg/mL) with the use of insulin (100 nM) as a positive control, with or without 30 min pretreatment with 100nM wortmannin (**A**), 1 µM GSK690693 (**B**), 5 µM Akt inhibitor VIII (**C**), 50 µm PD 98059 (**D**), or 10 µM SB 2035810 (**E**). The uptake of [3H]2-Deoxy-D-glucose into cells was determined as described in Materials and Methods. Data are expressed as percent of basal uptake in control cells. The data represent the mean ± SE of measurement made on six replicates in each of at least four independent experiments. * *p* < 0.05, and *** *p* < 0.0005, analyzed by one-way ANOVA, followed by Bonferroni’s post-hoc testing.

In order to clarify the role of PI3K-Akt pathway, as well as MAPK pathway, in the induction of SSE-dependent glucose uptake, selective inhibitors were used. PI3K, the upstream activator of Akt, was inhibited by wortmannin, a selective and irreversible inhibitor of the kinase. The stimulatory effect of insulin and SSE on glucose uptake was completely blocked by wortmannin (Figure 5A), suggesting that PI3K activity is required for SSE function.

Inhibition of Akt by GSK690693, an ATP competitive inhibitor, completely blocked glucose uptake induced by both insulin and SSE (Figure 5B). This effect was also found using an additional inhibitor, Akt inhibitor VIII, which is a pleckstrin homology (PH) domain-dependent inhibitor of Akt phosphorylation and activation (Figure 5C) [40]. These results suggest that although not significantly phosphorylated, Akt is crucial for the induction of glucose uptake by SSE.

The role of MAPK pathway in the induction of SSE-dependent glucose uptake was also investigated. Inhibition of p38 and MEK1/2 by SB2035810 and PD98059, respectively, exhibited some inhibition of glucose uptake that did not reach a statistical significance (Figure 5D,E).

**Figure 6:**
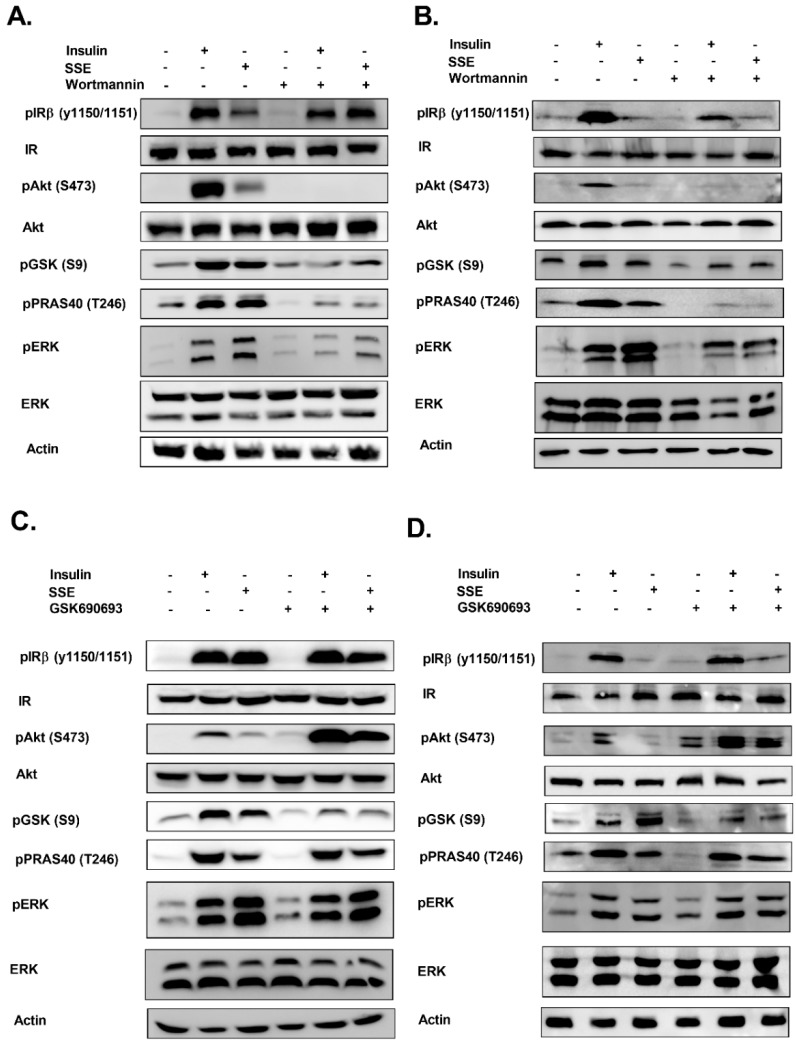
Role of PI3K and Akt in SSE-dependent phosphorylation of signaling proteins. L6 myotubes and 3T3-L1 adipocytes were treated with wortmannin (100 nM) (**A**,**B**, respectively) or GSK690693 (1 µM) (**C**,**D**, respectively), as indicated, for 30 min followed by additional 10 or 30 min treatment (in L6 and 3T3-L1, respectively) with insulin or SSE. Western blot analysis was performed as described in the Methods Section. These are representative results of three independent experiments.

We also followed the role of PI3K-Akt in the phosphorylation of PRAS40 and GSK3β, which were phosphorylated by SSE. This was measured in both L6 myotubes (Figure 6A) and adipocytes (Figure 6B). Inhibition of PI3K blocked insulin-dependent phosphorylation of Akt, as expected. In addition, wortmannin inhibited both insulin and SSE-induced phosphorylation of GSK3β and PRAS40. Blockade of PI3K activity partially inhibited ERK phosphorylation as well.

Akt inhibition by GSK690693 enhanced its phosphorylation in the control and treated cells, in accordance with previous reports, demonstrating that inhibition of the ATP binding site blocked Akt activation, while increasing its phosphorylation, as a result of a reduced accessibility of phosphatases to the phosphorylated site [41]. GSK690693 reduced insulin and SSE-dependent phosphorylation of GSK3β (Figure 6C,D), while PRAS40 phosphorylation was not affected. ERK phosphorylation was not altered by the inhibition of Akt. IR phosphorylation was not affected in most experiments.

## 4. Discussion

Insulin resistance is the primary pathogenic process underlying T2DM. In addition to its critical role in the development of the disease, insulin resistance has wide ranging consequences, and is considered as the underlying pathological mechanism of several other disorders such as poly cystic ovary syndrome, non-alcoholic fatty liver diseases, as well as dementia and Alzheimer disease [42,43]. Accordingly, therapeutic strategies targeted to improve insulin sensitivity, or to mimic its action, might be preferred in the treatment of T2DM and its complications over hypoglycemic drugs that act via other mechanisms [44]. Our study, demonstrating the activation of insulin signaling cascade by a botanical preparation of *Sarcopoterium spinosum* root extract, is important in view of the search for new agents to fight insulin resistance.

This study provides an additional support to our previous observations, demonstrating that SSE exerts insulin-like effects in vitro [29,31] and improves glucose tolerance and insulin sensitivity in HFD-fed mice and KK-Ay diabetic mice [29,31,32]. In addition, although not completely solved, this study sheds additional light on the mechanism of action mediating the activation of insulin signaling by SSE and supports the role of the PI3K-Akt pathway in SSE action. Additional in vivo mechanistic investigations involving the use of “omics” technologies [45] can identify the metabolite changes and might promote our understanding of the pathways involved in SSE effects.

In this study, the traditional use of SSE for the treatment of diabetes was confirmed by a hypothesis-free approach of high throughput serine-threonine phosphoprotein analysis, demonstrating that SSE activates the insulin signaling cascade. These results are also important in view of toxicological aspects; the results of this experiment, suggesting the lack of activation of unrelated pathways, indicate a specific biological activity of the active component/s of SSE.

The additive effect of SSE over insulin action indicates that, although insulin signaling is activated by SSE, there should be some differences, presumably in upstream events between SSE and insulin activation of this pathway. It might be hypothesized that subjects with defects in the activation of upstream events in insulin signaling might benefit from an agent that enables the activation of the insulin pathway through different mechanisms of action other than insulin. The hypothesis that SSE utilized different upstream mechanisms to activate downstream events was further supported by our results, demonstrating that maximal efficiency of glucose uptake was achieved by either insulin or SSE during different stages of differentiation. This indicates that SSE induces glucose uptake by recruiting similar, but not identical, players compared to insulin.

**Figure 7:**
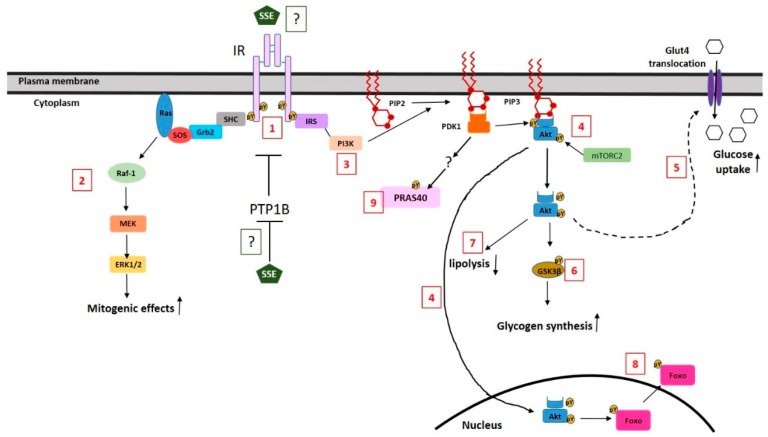
A schematic illustration of the effects of SSE on insulin signaling and the suggested mechanism. SSE induced a low level of IR phosphorylation (1). This phosphorylation might be the result of phosphatase inhibition or a direct binding and activation of the receptor by SSE. SSE activates MAPK pathway in a similar time course as induced by insulin (2). SSE activates the PI3K-Akt pathway (3); although a low level of Akt phosphorylation is detected, Akt is translocated to the membrane and the nucleus (4). Metabolic effects of the PI3K-Akt pathway are regulated by SSE, similar to insulin effects; glucose uptake (5) is increased via Glut4-facilitated transport, while GSK3β is phosphorylated, indicating increased glycogen synthesis (6) and lipolysis inhibition as well (7). In addition, FoxO is phosphorylated and is excluded from the nucleus (8). PRAS40 is also phosphorylated following insulin and SSE stimulation in a PI3K-dependent, Akt-independent mechanism. The scheme is based on results of this study and on our previous data [29,31].

A large number of natural products has been suggested to improve glycemic control in T2DM, mediated by several different mechanisms of action. Inhibition of carbohydrate digestion and absorption in the intestine, stimulating insulin secretion and reducing the efficacy of glucose re-absorption in the kidney, are some of these mechanisms [46]. However, as insulin resistance of target tissues is the major disturbance in T2DM, insulin sensitizing or insulin mimetic compounds might be preferred. Some natural compounds induced glucose transport via the activation of AMPK, which enables an insulin-independent pathway for glucose transport. Other natural products, which exert an insulin-sensitizing effect, act by ameliorating pathologies that inhibit insulin signaling, such as oxidative stress and inflammation. Other compounds activate insulin signaling either through the activation of PPARγ, increasing the expression of key signaling proteins, or directly by positively regulating PI3K-Akt pathway, acting as the direct agonist of the receptor [47] or by activating downstream components of the signal [46,48]. Our results from this and from our previous in vitro studies, negate the role of AMPK in mediating SSE effects [31,34] and suggest that SSE mimic insulin action, leading to a rapid activation of insulin signaling. A scheme summarizing SSE effects on insulin signaling is presented in Figure 7.

Although some phosphorylation of IR was detected following SSE treatment, the level and the time course of this phosphorylation suggest that this event might be the result of indirect induction of IR phosphorylation via phosphatase inactivation by SSE. Inhibition of tyrosine phosphatases, such as PTP1B and TCPTP, was found to stimulate insulin signaling and glucose uptake [49,50] and to ameliorate insulin resistance in diabetic mice [51]. Phosphatase inhibition by natural compounds, such as ursolic and oleanolic acids, was found to strengthen insulin effects [52,53,54], similar to the additive effect of SSE and insulin found in this study. The possible effect of SSE on such phosphatases and the role of such putative inhibition in mediating SSE effects should be investigated further. Still, we cannot exclude the possibility that SSE acts as a direct ligand for IR, enabling a minor, but physiologically significant phosphorylation of IR and Akt, which might be sufficient to stimulate the downstream effectors which amplifies and intensifies the signal further to enable the physiological response.

Our study demonstrates the activation of both the MAPK pathway and the PI3K-Akt pathway by SSE. ERK phosphorylation by SSE was demonstrated here for the first time, indicating that in addition to its insulin-like metabolic effect, SSE also activates the mitogenic pathway of MAPK. The insulin signaling cascade diverges to the activation of MAPK at the level of the receptor itself, or its substrate IRS, that serves as the docking site for Shc and Grb2, respectively [14]. Thus, activation of both major pathways of insulin by SSE suggests that the active component of the extract acts at the upstream part of insulin signaling. However, it should be noted, that these two pathways are not simple linear ones, and a crosstalk exists between them, enabling the activation of either MAPK pathway by the PI3K pathway, and vice versa [55,56]. This was also shown by our results, demonstrating reduced Erk phosphorylation upon PI3K inhibition.

We found that the PI3K-Akt pathway is required to induce glucose uptake by SSE. The finding, demonstrating that inhibition of PI3K by wortmannin completely abrogated SSE effects on glucose uptake, is in conflict with our previous publication [31], in which LY294002 failed to block SSE-induced glucose uptake. This contradiction might be the result of the different mechanisms of PI3K inhibition by wortmannin and LY294002. While wortmannin forms covalent and irreversible interactions with lysine residue in the ATP-binding site of p110 kinase domain of PI3K, preventing ATP binding and kinase activity [57], LY294002 is a reversible competitor of ATP binding [58]. The difference between these two results suggests that SSE includes or induces the production of some metabolites that compete with LY294002. This hypothesis should be further investigated.

Inhibition of Akt action by two different inhibitors completely blocked SSE-induced glucose uptake and GSK3β phosphorylation. It was also previously presented that SSE induced the translocation of Akt to the membrane and the nucleus [31]. However, our results show that Akt was barely phosphorylated on its S473 residue following SSE treatment. Moreover, the minor phosphorylation that was found occurred later than expected compared to the time course of GSK3β phosphorylation, which is a substrate of Akt. Thus, while Akt activation is crucial for the induction of SSE effects, the role of AKT phosphorylation in this Akt-mediated activity should be clarified further. It was already reported, that while Akt S473 phosphorylation is a biomarker for Akt activation, the actual importance of this event for the activation of Akt is under controversy [5,59,60,61].

Our study demonstrates the activation of glucose uptake and phosphorylation of GSK3β by SSE in the PI3K-Akt dependent mechanism. PRAS40 was also phosphorylated by both insulin and SSE. Activation of PI3K pathway induces PRAS40 phosphorylation and dissociation from mTORC1, enabling protein translation and cell growth [62,63]. Surprisingly, while PI3K inhibition by wortmannin abrogated insulin and SSE-induced phosphorylation of PRAS40, Akt inhibition did not affect this event. This is in contradiction to the recognition of PRAS40 as the Akt substrate [64]. Interestingly, in their study, Rhodes et al. investigated the effect of the Akt inhibitor GSK690693 on Akt phosphorylation and on its putative substrates in BT474 cells, demonstrating that while IC_50_ for Akt inhibition was around 10 nM, a much higher concentration of the inhibitor (>100 µM) was required to inhibit PRAS40 phosphorylation. This huge difference in IC50 was not explained by authors and is worth additional study. Our results support the role of PI3K in mediating the effect of SSE on PRAS40 phosphorylation as well as other downstream effects such as GSK3β phosphorylation and glucose uptake.

The complete chemical analysis of SSE composition is still missing, and a description of the known components and their potential role in mediating the anti-diabetic activity of SSE was reviewed by us before [64]. In short, we have already identified catechins and epicatechins in SSE, and the existence of a dimeric form of catechins was suggested [29,34]. In addition, several pentacyclic triterpenoids were identified in SSE [39], mainly ursolic and tormentic acid. However, it remains unclear whether these compounds are the active components in SSE. At the moment, we have an ongoing study aimed to isolate and identify the active compound/s in SSE.

## 5. Conclusions

In conclusion, our findings demonstrate that SSE activates the insulin signaling cascade and highlight its potential as a novel botanical drug for the treatment of insulin resistance. Isolation of active compounds and their identification is currently under investigation and might enable the development of new drugs for the treatment of T2DM.

## Figures and Tables

**Table 1:** Pathways significantly affected by SSE in 3T3-L1 adipocytes.

Top Canonical Pathways Activated by SSE	*p* Value
Insulin receptor signaling	5.58 × 10^−8^
ERK/MAPK signaling	9.67 × 10^−7^
AMPK signaling	1.18 × 10^−5^
mTOR signaling	2.12 × 10^−5^
14-3-3 mediated signaling	3.01 × 10^−5^
Acute Myeloid Leukemia Signaling	1.82 × 10^−4^
Superpathway of Inositol Phosphate Compounds	1.05 × 10^−3^
Signaling of Rho family GTPases	1.80 × 10^−3^
3-phosphoinositide Biosynthesis	1.83 × 10^−3^
Regulation of eIF4 and p70S6K signaling	2.71 × 10^−3^

3T3-L1 adipocytes at the 14th day of differentiation were treated with SSE (70 µg/mL) for 30 min. Phosphoproteomic analysis was performed as described in Materials and Methods. The top 10 pathways predicted by Ingenuity Pathway Analysis (CST) to be activated by SSE in 3T3-L1 adipocytes are presented. ERK, Extracellular signal-regulated kinases, MAPK: Mitogen activated protein kinase, AMPK: AMP-activated kinase, mTOR: mammalian target of rapamycin.

**Table 2:** Pathways significantly affected by insulin in 3T3-L1 adipocytes.

Top Canonical Pathways Activated by Insulin	*p* Value
Insulin receptor signaling	1.17 × 10^−5^
AMPK signaling	3.73 × 10^−5^
Glucocorticoid receptor signaling	5.23 × 10^−4^
Estrogen receptor signaling	8.16 × 10^−4^
Actin cytoskeleton signaling	8.31 × 10^−4^
mTOR signaling	1.3 × 10^−3^
Acute Myeloid Leukemia Signaling	2.6 × 10^−3^
RhoA Signaling	3.47 × 10^−3^
Superpathway of Inositol Phosphate Compounds	3.57 × 10^−3^
PPAR Signaling	3.57 × 10^−3^

3T3-L1 adipocytes at the 14th day of differentiation were treated with insulin (100 nM) for 30 min. Phosphoproteomic analysis was performed as described in Materials and Methods. The top 10 pathways predicted by Ingenuity Pathway Analysis (CST) to be activated by insulin in 3T3-L1 adipocytes are presented. AMPK: AMP-activated kinase, mTOR: mammalian target of rapamycin. PPAR: Peroxisome proliferator activated receptor.

## Supplementary Materials

The following are available online at https://www.mdpi.com/2072-6643/11/6/1396/s1, Table S1: Phosphopeptides regulated by SSE and insulin, Table S2. Phosphopeptides regulated by SSE, but not affected by insulin, Table S3. Phosphopeptides regulated by insulin, but not affected by SSE.
