# Supplementary material for: Activation of Insulin Signaling in Adipocytes and Myotubes by Sarcopoterium Spinosum Extract
Source: Nutrients. 2019 Jun 21;11(6):1396. doi: 10.3390/nu11061396 (PMC6628217; doi:10.3390/nu11061396)
Supplement: Supplementary file 1 [file nutrients-11-01396-s001.pdf]

Table S1. Phosphopeptides regulated by SSE and insulin.

Phospho Site	Entrez Gene Name	ID	Phospho Fold Change	p-value	Regulation by SSE	Regulation by insulin
AAADDGEEPKS(ph)EPETK	apurinic/apyrimidinic endodeoxyribonuclease 1	P28352	387156.5	1.31E-07	upregulation	upregulation
AAADDGEEPKS(ph)EPETKK	apurinic/apyrimidinic endodeoxyribonuclease 1	P28352	1746780.125	2.46E-09	upregulation	upregulation
AEDGAAPS(ph)PSSET(ph)PKKK	myristoylated alanine rich protein kinase C substrate	P26645	361423.656	1.21E-05	upregulation	upregulation
AEDGAAPSPSSET(ph)PKK	myristoylated alanine rich protein kinase C substrate	P26645	1388763.5	3.18E-06	upregulation	upregulation
AEGSLHSS(ph)PAGPSSSK	zinc finger CCCH-type containing 4	Q6ZPZ3	233355.797	3.37E-07	upregulation	upregulation
AENQRPAEDSALS(ph)PGPLAGAK	LRR binding FLII interacting protein 1	Q3UZ39	200312.812	8.39E-06	upregulation	upregulation
AHTDEPHS(ph)PQSKK	elaC ribonuclease Z 2	Q80Y81	270994.781	1.02E-05	upregulation	upregulation
ALRPS(ph)HR	SUMO1/sentrin/SMT3 specific peptidase 3	Q9EP97	534423.875	1.47E-06	upregulation	upregulation
ALSRS(ph)NEQMVSEKPSESK	calpastatin	P51125	84.676	0.0369	upregulation	upregulation
APLKDEQEMRAS(ph)PK	MYC binding protein 2, E3 ubiquitin protein ligase	Q7TPH6	577790.75	2.11E-08	upregulation	upregulation
AQGKPVSGQESSQS(ph)PYER	SEC31 homolog A, COPII coat complex component	Q3UPL0	459532.938	6.81E-10	upregulation	upregulation
AQQPAPASS(ph)PVKR	megakaryoblastic leukemia (translocation) 1	Q8K4J6	297751.812	9.66E-07	upregulation	upregulation
ARNDESQLS(ph)PATR	plectin	Q9QXS1	309582.156	6.65E-10	upregulation	upregulation
AS(ph)PGGVSTSSSDGKAEK	atrophin 1	O35126	121647.516	3.01E-07	upregulation	upregulation
ATAPQTQHVS(ph)PMR	eukaryotic translation elongation factor 1 delta	P57776	2.008	0.0181	upregulation	upregulation
AYRPPS(ph)GEK	natural killer cell triggering receptor	P30415	287889.656	2.13E-07	upregulation	upregulation
DDTSRYDERPGPS(ph)PLPHR	small nuclear ribonucleoprotein U1 subunit 70	Q62376	2.187	0.0473	upregulation	upregulation
DGLPRTPS(ph)R	serine/arginine repetitive matrix 2	Q8BTI8	187240.641	5.63E-07	upregulation	upregulation
DKPRS(ph)PFSK	RAB11 family interacting protein 5	A0A0N4SW73	322016.312	1.11E-06	upregulation	upregulation
EAGS(ph)EAESSSADPGPGRK	ribosomal RNA processing 1 homolog (S. cerevisiae)	P56183	149069.281	3.31E-07	upregulation	upregulation

EFKENKEPS(ph)PK	myosin IXA	Q8C170	802283.875	4.92E-08	upregulation	upregulation
EFTGS(ph)PPPSATKK	TBC1 domain family member 5	Q80XQ2	2.928	0.0101	upregulation	upregulation
EGLS(ph)PAKR	pre-mRNA processing factor 4B	Q61136	748079.312	1.66E-06	upregulation	upregulation
EIKPSEKPVS(ph)PK	Rho guanine nucleotide exchange factor 7	Q9ES28	1.978	0.0284	upregulation	upregulation
EKENGFSS(ph)PPR	DNA topoisomerase I	Q04750	753371.25	1.01E-07	upregulation	upregulation
ERSVS(ph)VDSGEQR	B cell CLL/lymphoma 9 like	Q67FY2	353553.156	2.51E-07	upregulation	upregulation
ERTSS(ph)LTHSEEK	RAN binding protein 3	Q9CT10	133.649	0.0407	upregulation	upregulation
ESS(ph)PSHGLLK	valosin containing protein interacting protein 1	Q8CDG3	623199	6.42E-08	upregulation	upregulation
ETNVSKEDT(ph)DQEEKASNEDVTK	PC4 and SFRS1 interacting protein 1	Q99JF8	592878.875	2.13E-07	upregulation	upregulation
FGLHGS(ph)SGK	exocyst complex component 1	Q5PPR2	430030.375	5.97E-08	upregulation	upregulation
FT(ph)DTRKDEQER	eukaryotic translation elongation factor 2	P58252	1429910.125	4.31E-07	upregulation	upregulation
GGDGAPRGS(ph)PSPASVSSGR	A-Raf proto-oncogene, serine/threonine kinase	P04627	399218.875	7.62E-08	upregulation	upregulation
GGGQSS(ph)PQEEPTWKK	CWC22 spliceosome associated protein homolog	Q8C5N3	201069.719	8.51E-07	upregulation	upregulation
GGLPVPKKS(ph)PK	membrane associated guanylate kinase, WW and PDZ domain containing 1	Q6RHR9	236670.172	4.97E-07	upregulation	upregulation
GGSSEELHDS(ph)PR	HDGF like 2	Q3UMU9	570404.125	1.59E-08	upregulation	upregulation
GINGGPSRMS(ph)PK	ataxin 2 like	Q7TQH0	309132.188	5.56E-07	upregulation	upregulation
GINGGPSRMS(ph)PK	ataxin 2 like	Q7TQH0	1326632.5	4.17E-07	upregulation	upregulation
GIS(ph)SSNEGVEEPSK	CDKN2A interacting protein	Q8BI72	314004.75	1.88E-08	upregulation	upregulation
GKLQEEGGS(ph)EEEEAGNPSEDGMQSGPTQAPPR	PWP1 homolog, endonuclein	Q99LL5	92.275	0.0361	upregulation	upregulation
GRAS(ph)PGGVSTSSSDGK	atrophin 1	O35126	428015.312	6.49E-08	upregulation	upregulation
GREEDSS(ph)PGPQSQSHRT	zinc finger (CCCH type), RNA binding motif and serine/arginine rich 1	Q64707	425661.312	1.86E-07	upregulation	upregulation
GRTSST(ph)NEDEDLNPEQK	transcription factor 12	Q3UXQ3	295773.125	1.11E-09	upregulation	upregulation

GS(ph)PVTTREPTPR	nuclear receptor corepressor 2	Q9WU42	319346.562	8.14E-06	upregulation	upregulation
GSGRGGTFT(ph)APR	LSM14A, mRNA processing body assembly factor	Q8K2F8	106.212	0.0441	upregulation	upregulation
GSPHSEGGKRS(ph)PEPSK	nuclear receptor corepressor 2	Q9WU42	190097.172	1.09E-06	upregulation	upregulation
GTEKRES(ph)PSPAPKPR	serine/arginine repetitive matrix 1	Q52KI8	2915868.75	3.27E-07	upregulation	upregulation
GTSDSSSGNVS(ph)EGDSPPDSQEDTFHGR	PHD finger protein 10	Q9D8M7	206119.656	1.32E-07	upregulation	upregulation
GYPSPGAHS(ph)PR	tensin 2	Q8CGB6	347042.156	3.12E-08	upregulation	upregulation
HASSS(ph)PESLKPTPAPGSR	serine/arginine repetitive matrix 2	Q8BTI8	228375.859	2.23E-07	upregulation	upregulation
HSSET(ph)FSAPTR	insulin receptor substrate 1	P35569	1220937.375	5.75E-07	upregulation	upregulation
HTAPSS(ph)PSPGTRT	transforming growth factor beta receptor associated protein 1	Q3UR70	745755.688	4.63E-07	upregulation	upregulation
IDASKNEEDEGHSNS(ph)SPR	heterogeneous nuclear ribonucleoprotein D	Q60668	17701528	1.06E-05	upregulation	upregulation
IPRPSVSQGCS(ph)R	cytoplasmic linker associated protein 2	Q8BRT1	535929.312	2.39E-07	upregulation	upregulation
ISQHGGS(ph)STSLSSTK	doublecortin like kinase 1	Q9JLM8	377344.531	2.07E-09	upregulation	upregulation
KAGS(ph)DAAASRPR	ribosomal protein L6	P47911	373325.688	3.67E-09	upregulation	upregulation
KEVQS(ph)PEQVK	BCL2 associated transcription factor 1	Q8K019	126.77	0.0408	upregulation	upregulation
KEVQS(ph)PEQVKSEK	BCL2 associated transcription factor 1	Q8K019	2.462	0.0223	upregulation	upregulation
KLGVSVS(ph)PSR	zinc finger CCCH-type containing 18	Q0P678	484465.094	6.03E-07	upregulation	upregulation
KLRSPT(ph)K	Rho GTPase activating protein 29	Q8CGF1	2777825.5	5.77E-07	upregulation	upregulation
KNS(ph)FGYR	La ribonucleoprotein domain family member 4B	Q6A0A2	183152.531	5.94E-08	upregulation	upregulation
KPIKS(ph)PSKDASSGK	pre-mRNA processing factor 4B	Q61136	233662.266	7.83E-09	upregulation	upregulation
KPS(ph)PQPS(ph)PPR	cyclin K	O88874	2.063	0.0258	upregulation	upregulation
KQES(ph)PLLVTK	RUN and cysteine rich domain containing beclin 1 interacting protein	Q80U62	645577.562	1.69E-07	upregulation	upregulation
KRS(ph)PSPSPTPEAK	SWI/SNF related, matrix associated, actin dependent regulator of chromatin subfamily c member 2	Q6PDG5	485130.406	8.9E-08	upregulation	upregulation

KSLSDS(ph)ESDDSKSK	chromobox 3	P23198	501260.062	2.44E-07	upregulation	upregulation
KVAPLSSS(ph)LDTSLDFSK	UV radiation resistance associated	Q8K245	114872.156	3.91E-07	upregulation	upregulation
KVELS(ph)ESEEDKGSK	serine/arginine repetitive matrix 1	A2A8V9	8.211	0.0173	upregulation	upregulation
LASDDRPS(ph)PPR	RNA binding motif protein 10	Q99KG3	1.636	0.035	upregulation	upregulation
LNHS(ph)PPQSSSR	RNA binding motif protein 26	Q6NZN0	487203.062	5.64E-08	upregulation	upregulation
LNRSDS(ph)DSSTLAK	WW and C2 domain containing 2	Q6NXJ0	1148141.375	5.87E-09	upregulation	upregulation
LPLEPETPGSLVGS(ph)PR	SZT2, KICSTOR complex subunit	A2A9C3	245182.984	7.84E-07	upregulation	upregulation
LRQQSSSSKGDS(ph)PELKPR	DAB2 interacting protein	Q3UHC7	702888.125	7.85E-08	upregulation	upregulation
LRS(ph)PSNDSAHR	zinc finger CCCH-type containing 13	E9Q784	911690.062	3.69E-07	upregulation	upregulation
LRTS(ph)PALK	centrosomal protein 170	Q6A065	363029.781	8.57E-07	upregulation	upregulation
LS(ph)PCLHR	family with sequence similarity 117 member A	Q7TNF9	412411.656	9.66E-09	upregulation	upregulation
LSSLEKSS(ph)PTPR	eukaryotic translation elongation factor 1 delta	P57776	893497	3.34E-07	upregulation	upregulation
LSSLRAS(ph)TSK	ribosomal protein S6	P62754	705318.375	1.4E-07	upregulation	upregulation
LSSLRAS(ph)TSKSESSQK	ribosomal protein S6	P62754	826636	2.3E-06	upregulation	upregulation
LSSS(ph)LDNKEK	protein phosphatase 1 regulatory subunit 12A	Q9DBR7	398166.219	6.89E-08	upregulation	upregulation
LSSSSS(ph)REPYK	YTH domain containing 1	E9Q5K9	288866.438	7.25E-10	upregulation	upregulation
LVEPHS(ph)PSPSSK	zinc finger protein 609	Q8BZ47	421858.531	1.25E-08	upregulation	upregulation
MSSEGPPRMS(ph)PK	ataxin 2	O70305	956224.312	2.25E-07	upregulation	upregulation
NEKS(ph)EEEQSSASVK	heterogeneous nuclear ribonucleoprotein C (C1/C2)	Q9Z204	161.239	0.0381	upregulation	upregulation
NEKS(ph)EEEQSSASVKK	heterogeneous nuclear ribonucleoprotein C (C1/C2)	Q9Z204	257446.891	4.48E-07	upregulation	upregulation
NGHS(ph)PERPTASDCQSPENSHDAGNCSNLMEETK	golgin A2	A2AN46	340863.062	4.06E-06	upregulation	upregulation
NNPKS(ph)PQKPIVR	B-Raf proto-oncogene, serine/threonine kinase	P28028	3.116	0.00764	upregulation	upregulation
NSQEDSEDS(ph)EEKDVK	nuclear casein kinase and cyclin dependent kinase substrate 1	Q80XU3	1999562	1.06E-05	upregulation	upregulation
QEPGGSHMS(ph)EDTEDTGR	G-patch domain containing 8	A2A6A1	261499.969	1.55E-08	upregulation	upregulation

QEQLS(ph)PR	nuclear receptor corepressor 2	F8VQL9	488683.25	1.27E-06	upregulation	upregulation
QGSGRES(ph)PSLVSR	ataxin 2 like	Q7TQH0	4.832	0.046	upregulation	upregulation
QKIDDRDS(ph)EEEGPSNQR	DEAD-box helicase 54	Q8K4L0	978283.562	8.37E-08	upregulation	upregulation
QQASKS(ph)PYNGVR	cyclin L1	Q52KE7	954296.25	1.29E-07	upregulation	upregulation
QSEES(ph)PADDGELRR	ATRX, chromatin remodeler	Q61687	1459684.25	2.92E-07	upregulation	upregulation
QSVDKVTS(ph)PTKV	caldesmon 1	D3Z6I7	614611.562	2.81E-07	upregulation	upregulation
QVQQNS(ph)LHR	intersectin 1	Q9Z0R4	218894.031	2.86E-07	upregulation	upregulation
RAKS(ph)PTPDGSER	YTH domain containing 1	E9Q5K9	223517.453	7.09E-07	upregulation	upregulation
RAS(ph)DGGANIQLHAQQLLK	SIK family kinase 3	Q6P4S6	3.684	0.0149	upregulation	upregulation
RAS(ph)SEIVTEGK	SUMO1/sentrin specific peptidase 7	Q8BUH8	1075709	1.78E-08	upregulation	upregulation
RAVVVS(ph)PKEENK	cyclin K	O88874	389618.062	1.94E-08	upregulation	upregulation
RDEQAAS(ph)DPMDQETVSR	MON2 homolog, regulator of endosome-to-Golgi trafficking	Q80TL7	258338.672	2.33E-07	upregulation	upregulation
RDS(ph)FSENEK	CWC22 spliceosome associated protein homolog	Q8C5N3	353478.688	1.65E-10	upregulation	upregulation
RDSSES(ph)QLASTESDKPTTGR	chromosome 18 open reading frame 25	Q8BH50	4.226	0.0259	upregulation	upregulation
RGFS(ph)DSGGGPPAK	pinin, desmosome associated protein	O35691	193.596	0.0322	upregulation	upregulation
RHS(ph)SPSSPTSPK	cyclin dependent kinase 14	O35495	360956.031	1.87E-08	upregulation	upregulation
RHSS(ph)PSSPTSPK	cyclin dependent kinase 14	O35495	145331.734	5.44E-08	upregulation	upregulation
RIQQQLGEEAS(ph)PR	Rho guanine nucleotide exchange factor 40	Q3UPH7	631480.812	7.81E-06	upregulation	upregulation
RKPS(ph)PEPEGEVGPPK	interferon regulatory factor 2 binding protein 2	E9Q1P8	2.636	0.036	upregulation	upregulation
RLPSSPAS(ph)PSPK	erythrocyte membrane protein band 4.1 like 1	A2AUK5	408303.594	4.76E-08	upregulation	upregulation
RLS(ph)PVEK	ETS variant 6	P97360	1745488.625	4.58E-09	upregulation	upregulation
RNS(ph)SSSSSPSERPR	echinoderm microtubule associated protein like 3	Q8VC03	908991.75	6.01E-08	upregulation	upregulation
RPAS(ph)VSSAAAEHEAR	interferon regulatory factor 2 binding protein 2	E9Q1P8	302072.375	5.93E-06	upregulation	upregulation

_RPGS(ph) VSSTDQER _	interferon regulatory factor 2 binding protein like	Q8K3X4	759844.688	1.65E-08	upregulation	upregulation
RPMEED GEEKS(ph) PSK	interleukin enhancer binding factor 3	Q9Z1X4	369781.938	1.86E-06	upregulation	upregulation
RPMEED GEEKS(ph) PSK	interleukin enhancer binding factor 3	Q9Z1X4	660405.625	6.25E-08	upregulation	upregulation
RQDTTRS (ph)PSLAP TQR	tensin 2	Q8CGB6	537194.875	1.5E-08	upregulation	upregulation
RQMS(ph) LTEK	DAB2 interacting protein	Q3UHC7	274077.562	9.87E-06	upregulation	upregulation
RSEAEEG EVRT(ph)P TK	erythrocyte membrane protein band 4.1 like 1	A2AUK5	981435.625	4.4E-07	upregulation	upregulation
RVSSET(p h)HQGPGT PESK	nuclear mitotic apparatus protein 1	E9Q7G0	278795.438	2.19E-05	upregulation	upregulation
RYS(ph)G DSDSSASS AQSGPMG AR	growth arrest specific 2 like 1	Q8JZP9	271003.688	3.37E-09	upregulation	upregulation
S(ph)NEQ MVSEKPSE SK	calpastatin	P51125	746087.062	1.51E-08	upregulation	upregulation
S(ph)PVK QDKSEIST DPK	serine/arginine repetitive matrix 2	Q8BTI8	158.494	0.0403	upregulation	upregulation
S(ph)QPC VLNDKK	family with sequence similarity 53 member B	Q8BGR5	572986	6.91E-08	upregulation	upregulation
S(ph)VSTL RPCAK	MON2 homolog, regulator of endosome-to-Golgi trafficking	Q80TL7	728687.312	5.01E-08	upregulation	upregulation
SAS(ph)E PSLHR	A-Raf proto-oncogene, serine/threonine kinase	P04627	3.845	0.0468	upregulation	upregulation
SAVRPSP S(ph)PER	serine/arginine repetitive matrix 2	Q8BTI8	1239224.5	2.6E-07	upregulation	upregulation
SCS(ph)FS SESR	DENN domain containing 4C	A6H8H2	438.305	0.0173	upregulation	upregulation
SES(ph)LS NCSIGKK	ankyrin repeat and sterile alpha motif domain containing 1A	P59672	3.275	0.0214	upregulation	upregulation
SGGPNSC KSDDYMP MS(ph)PTS VSAPK	insulin receptor substrate 2	P81122	262424.625	5.43E-06	upregulation	upregulation
_SGSS(ph)P EMKDKPR _	serine/arginine repetitive matrix 2	Q8BTI8	1559911.375	1.29E-07	upregulation	upregulation
SGSSQEL DGKPSAS(ph)PQER	serine/arginine repetitive matrix 2	Q8BTI8	285619.125	1.07E-08	upregulation	upregulation
SHLETM GSS(ph)PL STTK	serine/threonine kinase 11 interacting protein	Q3TAA7	325137.719	8.97E-07	upregulation	upregulation
SKPNVPA ES(ph)R	serine and arginine rich splicing factor 4	Q8VE97	307138.5	4.02E-16	upregulation	upregulation
SKS(ph)E DMDSVES K	activating transcription factor 7 interacting protein	Q7TT18	1128908.25	7.85E-11	upregulation	upregulation
_SKS(ph)Q SSGSSATH	insulin receptor substrate 2	P81122	3.228	0.0121	upregulation	upregulation

PISVPGAR_						
SPERTEEVLSPDGSPSKSPS(ph)K	adducin 3	Q9QYB5	1222310	1.93E-05	upregulation	upregulation
SPQESTGDPGNSSSVSDGKGSSERGS(ph)PIEK	transcriptional repressor GATA binding 1	Q925H1	289015.812	4.58E-07	upregulation	upregulation
SRS(ph)PFASTR	praja ring finger ubiquitin ligase 1	O55176	1122411.875	6.78E-08	upregulation	upregulation
SRTGS(ph)ESSQTGASATSGR	eukaryotic translation initiation factor 4B	Q8BGD9	4722655	3.6E-08	upregulation	upregulation
SRTPS(ph)ASHEEQQE	small glutamine rich tetratricopeptide repeat containing alpha	Q8BJU0	160.233	0.0418	upregulation	upregulation
SS(ph)FSNSADDIKSK	chromobox 5	Q61686	153437.078	1.08E-06	upregulation	upregulation
SS(ph)SKDSRPSQAAGDNQGDEAK	thyroid hormone receptor associated protein 3	Q569Z6	261790.469	1.16E-06	upregulation	upregulation
SS(ph)SSAGSEVGGQSTGSNHK	trinucleotide repeat containing 6B	Q8BKI2	307479.812	3.61E-07	upregulation	upregulation
SSESVVDEDGGRS(ph)PR	microtubule associated serine/threonine kinase 3	Q3U214	177248.125	1.23E-08	upregulation	upregulation
SSS(ph)LPSDRGPPAR	DENN domain containing 4C	A6H8H2	1.976	0.0301	upregulation	upregulation
STS(ph)LNERPK	TSC complex subunit 2	Q61037	927394.688	4.08E-06	upregulation	upregulation
SVGKVEPSSQSPGRS(ph)PR	ELKS/RAB6-interacting/CAST family member 1	Q99MI1	4.325	0.00251	upregulation	upregulation
T(ph)AEEEDEADPKR	parathymosin	Q9D0J8	275527.688	1.27E-07	upregulation	upregulation
T(ph)ASNAEQYKYGK	actin filament associated protein 1	Q80YS6	235155.312	0.000121	upregulation	upregulation
T(ph)KSDESGEEKNGDEDCQR	La ribonucleoprotein domain family member 1	Q6ZQ58	291132.531	1.6E-07	upregulation	upregulation
TAS(ph)EGSEAETPEAPKQPAKK	La ribonucleoprotein domain family member 7	Q05CL8	1317469.25	1.63E-05	upregulation	upregulation
TAVSVAQGGHS(ph)R	PBX homeobox 2	O35984	267831.438	7.98E-09	upregulation	upregulation
TDGS(ph)IS(ph)GDRQPVTVADYISR	FMR1 autosomal homolog 2	Q6P5B5	-8.626	0.0219	downregulation	upregulation
TDSEKPFRGSQS(ph)PK	thyroid hormone receptor associated protein 3	Q569Z6	69.604	0.0481	upregulation	upregulation

TEEVLSPDGSPSKS(ph)PSKK	adducin 3	Q9QYB5	2.621	0.0115	upregulation	upregulation
TFS(ph)ATVR	calcium regulated heat stable protein 1	Q9CR86	4.313	0.0329	upregulation	upregulation
TGS(ph)NISGASSDVSLDEQYKHQLEETK	oxysterol binding protein	Q3B7Z2	174067.953	2.85E-07	upregulation	upregulation
TGS(ph)TGALGPSER	protein phosphatase 1 regulatory subunit 12C	Q3UMT1	594034.25	5.57E-06	upregulation	upregulation
TKEYVSNDATQS(ph)DDEEKLQSQQTDTDGGR	WD repeat domain 44	Q6NVE8	3.289	0.0233	upregulation	upregulation
TS(ph)SDPNLNNHSQEVR	myotubularin related protein 4	Q91XS1	392583.156	1.22E-07	upregulation	upregulation
TSS(ph)DPNLNNHSQEVR	myotubularin related protein 4	Q91XS1	678778	2.91E-07	upregulation	upregulation
TSS(ph)TNEDEDLNPEQKIER	transcription factor 12	Q3UXQ3	216524.125	8.31E-07	upregulation	upregulation
TTS(ph)PTAESVRPTESMRR	lipase E, hormone sensitive type	P54310	3.647	0.0402	upregulation	upregulation
TVIRLPSGSGPAS(ph)PTTGSAVDIR	AHNAK nucleoprotein	E9Q616	2.498	0.0328	upregulation	upregulation
VALGDGVQLPPGDY(ph)STTPGGTLFSTT(ph)PGGTR	eukaryotic translation initiation factor 4E binding protein 1	Q60876	3.14	0.0326	upregulation	upregulation
VCS(ph)PYNHR	TOP1 binding arginine/serine rich protein	Q80Z37	274834.844	8.03E-08	upregulation	upregulation
VEPSSQSPGRS(ph)PR	ELKS/RAB6-interacting/CAST family member 1	Q99MI1	2486671	6.15E-08	upregulation	upregulation
VEQATKPS(ph)FESGR	RNA binding motif protein, X-linked like-1	Q91VM5	262625.688	4.51E-06	upregulation	upregulation
VKTVIS(ph)PR	serine/arginine repetitive matrix 2	Q8BTI8	2.175	0.0352	upregulation	upregulation
VLAPCS(ph)PSEERR	inositol-trisphosphate 3-kinase B	B2RXC2	1067020.875	3.8E-08	upregulation	upregulation
VLS(ph)PPHTK	ubiquitin associated protein 1	Q8BH48	184638.141	1.91E-09	upregulation	upregulation
VPS(ph)PDHRR	pre-mRNA processing factor 38A	Q4FK66	819750.938	4.24E-08	upregulation	upregulation
VRAHS(ph)PAEGASSESSSPGPK	folliculin	Q8QZS3	417612.906	1.77E-07	upregulation	upregulation
VRHDT(ph)PDPS(ph)PPR	BUD13 homolog	Q8R149	281939.938	1.5E-08	upregulation	upregulation

VSS(ph)ET HQGPGTP ESK	nuclear mitotic apparatus protein 1	E9Q7G0	726792.312	5.71E-10	upregulation	upregulation
_VTRS(ph) QEELREEK _	afadin, adherens junction formation factor	Q9QZQ1	866774.625	5.41E-05	upregulation	upregulation
VVSS(ph) TSEEEEAF TEK	oxidation resistance 1	E9Q0A7	235611.5	1.84E-06	upregulation	upregulation

Phosphopeptides regulated by both SSE and insulin. 3T3-L1 adipocytes at the 14th day of differentiation were treated with SSE (70 µg/ml) or insulin (100 nM) for 30 min. Phosphoproteomic analysis was performed as described in Materials and Methods. Only phosphopeptides that were significantly affected by both SSE and insulin are presented. P-value was calculated by Student's *t*-test, compared to control, untreated cells.

Table S2. Phosphopeptides regulated by SSE, but not affected by insulin.

Phospho Site	Entrez Gene Name	ID	Phospho Fold Change	*p*-value	Regulation by SSE
AGETRFT(ph)DTR	eukaryotic translation elongation factor 2	P58252	3017911.5	3.32E-05	upregulation
AQKPPS(ph)PPAMEN GTR	tRNA methyltransferase 1	Q3TX08	202794.172	1.66E-06	upregulation
DDSHSAEDS(ph)EDE KDDHK	nuclear casein kinase and cyclin dependent kinase substrate 1	Q80XU3	107450.258	4.99E-07	upregulation
EEALDEAEEPES(ph)P PPPPRS(ph)PS(ph)PEPT VVDTPSHASQSAR	arginine-glutamic acid dipeptide repeats	Q80TZ9	280205.812	9.97E-06	upregulation
EENVNS(ph)PEDKR	ATRX, chromatin remodeler	Q61687	252389.875	2.01E-06	upregulation
EEQSGPVDEKGNDS(p h)DGEAESDDPEKK	vacuolar protein sorting 4 homolog B	P46467	180176.672	2.37E-09	upregulation
EGKEDEAS(ph)TDVD EKPK	solute carrier family 16 member 1	P53986	175066.703	3.27E-09	upregulation
ELSSEPRT(ph)PPAQK	microtubule associated protein 1A	Q9QYR6	338646.125	0.000012	upregulation
ERGT(ph)PPVDPK	proline rich coiled-coil 2A	Q7TSC1	749880.562	9.82E-07	upregulation
ESEALPEKEGDELGEG ERPEDDTAAIELS(ph)S(ph)DEAVEVEEVIEESR	caveolae associated protein 1	O54724	-3.291	0.0406	downregulation
EYGS(ph)TSSIDR	signal induced proliferation associated 1 like 2	Q80TE4	281451.094	6.35E-06	upregulation
FHDS(ph)EGDDT(ph)E ETEDYR	BCL2 associated transcription factor 1	Q8K019	150250.812	2.42E-05	upregulation
FKAEAPLPS(ph)PK	AHNAK nucleoprotein	E9Q616	2.379	0.0338	upregulation
FLMECRNS(ph)PVAK	eukaryotic translation initiation factor 4E binding protein 1	Q60876	5.318	0.0374	upregulation
GAAAADLLSS(ph)SPE SQHGGTQPPGGGQPL LQPTK	protein tyrosine phosphatase, non-receptor type 23	Q6PB44	227383.828	8.7E-06	upregulation
GDS(ph)PELKPR	DAB2 interacting protein	Q3UHC7	485128.375	1.53E-05	upregulation
GEEGS(ph)EEEETENG PKPK	CTR9 homolog, Paf1/RNA polymerase II complex component	Q62018	545258.25	1.07E-07	upregulation
GGGSGGGDES(ph)EG EEVDED	purine rich element binding protein B	O35295	171745.594	0.000242	upregulation
GHYEVTGS(ph)DDEA GK	AHNAK nucleoprotein	E9Q616	548371.5	4.3E-06	upregulation

GLS(ph)VDSAQEVKR	transformation/transcription domain associated protein	Q80YV3	334797.375	3.74E-06	upregulation
HADGEKEDQFNGS(ph)PPRPQPR	vir like m6A methyltransferase associated	A2AIV2	2.345	0.039	upregulation
HSLDS(ph)DEEDDDEEGSSK	CD2 cytoplasmic tail binding protein 2	Q9CWK3	404969.844	7.38E-08	upregulation
IEDVGS(ph)DEEDDS(ph)GKDKK	heat shock protein 90 alpha family class B member 1	P11499	923927.812	5.22E-05	upregulation
ILEQQNSSRT(ph)LEK	septin 7	O55131	4.272	0.0118	upregulation
KEES(ph)EES(ph)DDDMGFGLFD	ribosomal protein lateral stalk subunit P2	P99027	-12.077	0.0376	downregulation
KPAATS(ph)PLSPMANGGR	pleckstrin homology like domain family B member 1	Q6PDH0	176993.266	8.66E-07	upregulation
KSRVS(ph)VS(ph)PGR	serine/arginine repetitive matrix 1	A2A8V9	1710918.125	0.000129	upregulation
KSS(ph)SSSGVPYSPAIPNK	exocyst complex component 7	O35250	258689.75	1.81E-06	upregulation
KTS(ph)GPPVSELITK	histone cluster 1, H1e	P43274	249619.781	6.98E-08	upregulation
LNHVAAGLVS(ph)PSLK	serine and arginine rich splicing factor 11	E9Q6E5	2.676	0.0246	upregulation
LRMS(ph)GAGK	phosphofructokinase, liver type	P12382	2379280.25	4.82E-05	upregulation
LVLS(ph)GEKK	eukaryotic translation initiation factor 4 gamma 3	Q80XI3	376698.531	0.000021	upregulation
MLAESDDS(ph)GDEESVSQTDKTELQSTLR	oxysterol binding protein	Q3B7Z2	175354.484	3.93E-06	upregulation
QASTDAGT(ph)AGALTPQHVR	Yes associated protein 1	P46938	2.845	0.0378	upregulation
QGS(ph)PTPALPEKR	tensin 1	A0A087WQ94	1.969	0.0471	upregulation
QGSGRESPS(ph)LVSR	ataxin 2 like	Q7TQH0	1496941.695	0.000071	downregulation
QQSHFAMMHGGTGFAGIDSSS(ph)PEVK	poly(rC) binding protein 1	P60335	3.988	0.0494	upregulation
RADANLLTDTGTESSPRSPVCS(ph)LR	exosome component 5	Q9CRA8	562552	4.89E-05	upregulation
RALS(ph)PLPAR	pleckstrin homology like domain family B member 1	Q6PDH0	3.358	0.0444	upregulation
RKAS(ph)GPPVSELITK	histone cluster 1 H1 family member c	P15864	280955.438	5.83E-08	upregulation
RKS(ph)ELGAEPGHFGVCVDSLTSDK	myosin IXB	Q9QY06	262473.25	5.8E-07	upregulation
RLS(ph)SLRAST(ph)SK	ribosomal protein S6	P62754	569575	0.000109	upregulation
RPPS(ph)PDPNTKVSEEAESQQWDTSK	spectrin beta, non-erythrocytic 1	Q62261	199120.609	1.02E-05	upregulation
RRDS(ph)YYDR	transformer 2 alpha homolog	A0A0N4SVC2	342607.469	4.61E-06	upregulation
RS(ph)PERPTGDLR	zinc finger CCCH-type containing 13	E9Q784	474525.812	9.34E-06	upregulation
RTAS(ph)AGTVSDAEAR	Ras association (RalGDS/AF-6) and pleckstrin homology domains 1	F2Z3U3	826671.812	3.09E-07	upregulation
RVES(ph)EESGDEEGKK	protein phosphatase 1 regulatory subunit 7	Q3UM45	272204.688	6.4E-06	upregulation
RVESEES(ph)GDEEGKK	protein phosphatase 1 regulatory subunit 7	Q3UM45	764244.938	6.69E-07	upregulation

RVQT(ph)PLLR	septin 9	Q80UG5	361650.531	5.79E-07	upregulation
S(ph)LPTSPERR	protein phosphatase 1 regulatory subunit 3D	A2AJW4	757086.625	3.45E-06	upregulation
SANDSTVHS(ph)PFTK	ubiquitin-associated protein 2-like	Q80X50	371178.438	5.96E-06	upregulation
SANQSPQSVGGS(ph)GIDS(ph)GVESTSDSLR	lipin 1	Q91ZP3	132761.469	2.33E-10	upregulation
SDDYMPMS(ph)PTSVSAPK	insulin receptor substrate 2	P81122	280300.531	9.2E-08	upregulation
SDDYMPMS(ph)PTSVSAPK	insulin receptor substrate 2	P81122	738699.25	0.000019	upregulation
SLPASGTPQS(ph)PPAVK	Ras association (RalGDS/AF-6) and pleckstrin homology domains 1	F2Z3U3	-2.337	0.0204	downregulation
SLPTS(ph)PERR	protein phosphatase 1 regulatory subunit 3D	A2AJW4	2.2	0.0303	upregulation
SPS(ph)ASSLSSMSSVASSVSSKPSR	CAP-Gly domain containing linker protein 1	Q922J3	-2.178	0.0356	downregulation
SQDATVSPGSEQSEKS(ph)PGPIVSR	zinc finger C3HC-type containing 1	Q80YV2	2.741	0.0188	upregulation
SQSRS(ph)HS(ph)PLPAPPSK	serine and arginine rich splicing factor 6	Q3TWW8	191331.281	2.11E-06	upregulation
SRTESITATS(ph)PASMVGGKPGSFR	insulin receptor substrate 1	P35569	256947.156	0.00003	upregulation
SSS(ph)PEDRYTEQER	CWC22 spliceosome associated protein homolog	Q8C5N3	140236.172	3.98E-08	upregulation
STPSQVTSEEKDGHS(ph)PMSK	microtubule associated protein 1A	Q9QYR6	961333.25	1.96E-06	upregulation
STSAPQMSPGS(ph)S(ph)DNQSSSPQPAQQK	ubiquitin-associated protein 2-like	Q80X50	100920.211	3.34E-09	upregulation
SVGKVEPSSQS(ph)PGR	ELKS/RAB6-interacting/CAST family member 1	Q99MI1	292814.812	1.23E-07	upregulation
SVTPDSLGHT(ph)PPAR	insulin receptor substrate 1	P35569	485900.844	1.37E-05	upregulation
TAS(ph)EGDGGAAGGAGTAGGRPMSVAGSPLSPGPVR	insulin receptor substrate 2	P81122	356408.75	1.64E-05	upregulation
TAS(ph)FSESRADEVAPAK	ATP citrate lyase	Q3TS02	4.264	0.0322	upregulation
TAS(ph)FSESRADEVAPAKK	ATP citrate lyase	Q3TS02	4.647	0.0112	upregulation
TDGFAEAIHS(ph)PQVAGVPR	translocated promoter region, nuclear basket protein	F6ZDS4	4.894	0.0295	upregulation
TEEVLSPDGSPSKS(ph)PSK	adducin 3	Q9QYB5	2.377	0.0435	upregulation
TEHAPS(ph)PSSGGTVK	cyclin dependent kinase 13	Q69ZA1	564.991	0.0146	upregulation
TELS(ph)PRPGAAGR	eukaryotic translation initiation factor 2B subunit delta	Q61749	137.318	0.0404	upregulation
TPLGAS(ph)LDEQSSGTPK	sperm specific antigen 2	Q922B9	2.02	0.0403	upregulation
VGGS(ph)SVDLHR	catenin delta 1	P30999	1048659.625	4.09E-05	upregulation
VPELDGAGSTEQDKSHS(ph)NSSTLSDR	Ral GTPase activating protein catalytic alpha subunit 2	A3KGS3	261927.562	9.38E-07	upregulation
VTNDSSSSSSSSSDSDS(ph)DGEEHDSDRAPR	NADH:ubiquinone oxidoreductase subunit V3	Q3U422	240584.797	2.84E-08	upregulation

YCRPESQEHPEADPG SAAPY(ph)LK	signal transducer and activator of transcription 3	P42227	3.175	0.041	upregulation

Phosphopeptides regulated by SSE, but not affected by insulin. 3T3-L1 adipocytes at the 14th day of differentiation were treated with SSE (70 µg/ml) or insulin (100 nM) for 30 min. Phosphoproteomic analysis was performed as described in Materials and Methods. Only phosphopeptides that were significantly affected by SSE, but not affected by insulin are presented. P-value was calculated by Student's *t*-test, compared to control, untreated cells.

Table S3. Phosphopeptides regulated by insulin, but not affected by SSE.

Phospho Site	Entrez Gene Name	ID	Phospho Fold Change	p-value	Regulation by Insulin
AASDGQYENQS(ph)PE ATSPR	tensin 1	A0A087W Q94	449598.5	0.0000206	upregulation
AAS(ph)DGQYENQSPE ATSPR	tensin 1	A0A087W Q94	464031.719	0.00105	upregulation
HAAYGGYST(ph)PEDR	tensin 1	A0A087W Q94	467941.969	0.0109	upregulation
RAAS(ph)DGQYENQSP EATSPR	tensin 1	A0A087W Q94	468104.031	0.0106	upregulation
EAFEEMEGTSPSS(ph)PP HSVAR	tensin 1	A0A087W Q94	468412.969	0.0114	upregulation
HPGAHQGNLASSLHSN AVISPGS(ph)PSLGR	tensin 1	A0A087W Q94	470820.656	0.0335	upregulation
IEQNLQS(ph)PTQQQTA R	unc-51 like autophagy activating kinase 1	A0A0R4J0 B3	596116.5	0.0453	upregulation
LPS(ph)SEIHPEESLYR	TBC1 domain family member 16	A2ABG4	374743.469	0.00123	upregulation
VQEESYPQS(ph)PR	BCLAF1 and THRAP3 family member 3	A2AG58	3.184	0.0163	upregulation
APS(ph)LTSDSEGKKPA QAVK	SEC16 homolog A, endoplasmic reticulum export factor	A2AIX1	183151.172	0.00549	upregulation
_SPDPEMVPRGS(ph)PVR _	SEC16 homolog A, endoplasmic reticulum export factor	A2AIX1	186046.203	0.00569	upregulation
YSEPERPSS(ph)R	SEC16 homolog A, endoplasmic reticulum export factor	A2AIX1	186616.578	0.0056	upregulation
RKPGAGGS(ph)PALAR	MAP7 domain containing 1	A2AJI0	8.099	0.0219	upregulation
TGS(ph)TSSKEDDYESD AATIVQK	ubiquitin protein ligase E3 component n-recognin 4	A2AN08	567648.562	0.0000707	upregulation
_TS(ph)PADHGGSVGSES GGSAVDSVAGEHSVSGR _	ubiquitin protein ligase E3 component n-recognin 4	A2AN08	568486.375	0.000214	upregulation
NGHSPERPTAS(ph)DC QSPENSHDAGNCSNLM EETK	golgin A2	A2AN46	5.145	0.0465	upregulation
HMSES(ph)PNRKVEK	peptidylprolyl isomerase G	A2AR02	25.727	0.0191	upregulation
KADREQS(ph)PVSK	peptidylprolyl isomerase G	A2AR02	28.84	0.00804	upregulation
QGDETPSTNNGS(ph)D DEKTGLK	RAB GTPase activating protein 1	A2AWA9	116371.164	0.000477	upregulation
IHRAS(ph)DPGLPAEEP KEKPPR	carbamoyl-phosphate synthetase	B2RQC6	3.377	0.0137	upregulation

	2, aspartate transcarbamylase, and dihydroorotase				
IHRAS(ph)DPGLPAEEPK	carbamoyl-phosphate synthetase 2, aspartate transcarbamylase, and dihydroorotase	B2RQC6	3.406	0.0205	upregulation
ARSPS(ph)PCPFR	inositol-trisphosphate 3-kinase B	B2RXC2	6.262	0.0314	upregulation
VTSSMSS(ph)PSMQPK	PTPRF interacting protein alpha 1	B2RXQ2	23.702	0.0115	upregulation
GAPHTVS(ph)HEDIR	PTPRF interacting protein alpha 1	B2RXQ2	25.155	0.0191	upregulation
CETS(ph)PPSS(ph)PRPLRLDR	PTPRF interacting protein alpha 1	B2RXQ2	25.651	0.00507	upregulation
DMS(ph)PTSTDTEVHR	AHNAK nucleoprotein 2	E9PYB0	2.167	0.0496	upregulation
RETVVESQSSQSPS(ph)PKR	SR-related CTD associated factor 11	E9PZM7	182249.141	0.00759	upregulation
DQKEEGNDYDT(ph)R	YTH domain containing 1	E9Q5K9	874268.5	0.049	upregulation
AEAPLPS(ph)PK	AHNAK nucleoprotein	E9Q616	1.966	0.0247	upregulation
ISMPDIDLHLKS(ph)PK	AHNAK nucleoprotein	E9Q616	2.035	0.0396	upregulation
GDLGASS(ph)PSMK	AHNAK nucleoprotein	E9Q616	2.11	0.00817	upregulation
LRS(ph)EDGVEGDLGETQSR	AHNAK nucleoprotein	E9Q616	2.133	0.0402	upregulation
SKGHYEVTGS(ph)DDEAGKLQGSGVSLASK	AHNAK nucleoprotein	E9Q616	2.149	0.0364	upregulation
VNGDDHHEEDMDMS(ph)D	serine and arginine rich splicing factor 11	E9Q6E5	336578.875	0.0135	upregulation
EEKRPTEAVS(ph)PK	serine and arginine rich splicing factor 11	E9Q6E5	338283.469	0.000262	upregulation
DYDEEEQGYDS(ph)EKEK	serine and arginine rich splicing factor 11	E9Q6E5	342376.344	0.00000744	upregulation
VNGDDHHEEDMDMS(ph)D	serine and arginine rich splicing factor 11	E9Q6E5	344838.094	0.000774	upregulation
VS(ph)SETHQGPGTPESK	nuclear mitotic apparatus protein 1	E9Q7G0	13.185	0.000992	upregulation
VSSETHQGPGT(ph)PESK	nuclear mitotic apparatus protein 1	E9Q7G0	13.375	0.00523	upregulation
VSS(ph)ETHQGPGTPESKK	nuclear mitotic apparatus protein 1	E9Q7G0	13.381	0.047	upregulation
LKQTENAFS(ph)PSR	caldesmon 1	E9QA16	3.452	0.0387	upregulation
DTTQRS(ph)PVHVQPTAR	AHNAK nucleoprotein 2	F7DBB3	2.192	0.0437	upregulation
GSPAPSSASSSASDLS(ph)RSPAHSR	G protein pathway suppressor 1	G3UXW9	5.21	0.0307	upregulation
YS(ph)PPRDDDKVDNQAK	thyroid hormone receptor interactor 12	G5E870	512973.469	0.00183	upregulation
TLS(ph)SSRPPLLLR	eukaryotic elongation factor 2 kinase	O08796	4.345	0.0244	upregulation
GRAS(ph)PGGVSTSSSDGKAEK	atrophin 1	O35126	2.725	0.028	upregulation
SSSAGKES(ph)PKVR	cyclin dependent kinase 14	O35495	3.797	0.0166	upregulation
RAQST(ph)DSLGTSSSLQSK	rabaptin, RAB GTPase binding effector protein 1	O35551	111418.531	0.0167	upregulation

GFS(ph)DSGGGPPAK	pinin, desmosome associated protein	O35691	22.659	0.00489	upregulation
SRPLS(ph)MDAR	serine/threonine kinase 10	O55098	354858.719	0.0158	upregulation
SPRPGSS(ph)VPEHSPR	unc-51 like autophagy activating kinase 1	O70405	606905.375	0.00144	upregulation
RMS(ph)LEGR	caspase 8	O89110	3.491	0.0491	upregulation
YSPTS(ph)PK	RNA polymerase II subunit A	P08775	23.113	0.000863	upregulation
EIS(ph)DDEAEEEKGEK	heat shock protein 90 alpha family class B member 1	P11499	5.726	0.0277	upregulation
IEDVGS(ph)DEEDDSGKDKK	heat shock protein 90 alpha family class B member 1	P11499	5.769	0.0124	upregulation
KVS(ph)ADGAAKAEPK	high mobility group nucleosome binding domain 1	P18608	5.399	0.0179	upregulation
SMS(ph)ADEDLQEPSR	negative elongation factor complex member E	P19426	12.199	0.00274	upregulation
RKS(ph)LSDSESDDSK	chromobox 3	P23198	3.581	0.0436	upregulation
SKVGS(ph)TENIK	microtubule associated protein 4	P27546	7.931	0.00432	upregulation
VGS(ph)TENIKHQPGGGR	microtubule associated protein 4	P27546	7.955	0.00402	upregulation
SKVGS(ph)TENIKHQPGGGR	microtubule associated protein 4	P27546	7.972	0.0127	upregulation
SLS(ph)QRSR	natural killer cell triggering receptor	P30415	12.532	0.000842	upregulation
EEQTDTS(ph)DGESVTHHIR	G protein nucleolar 1 (putative)	P36916	5.063	0.0273	upregulation
QIS(ph)EDVDGPDNR	neural precursor cell expressed, developmentally down-regulated 4	P46935	12.08	0.00301	upregulation
AGS(ph)DAAASRPR	ribosomal protein L6	P47911	143864.125	0.0494	upregulation
KEES(ph)EESEDDMGFGLFD	ribosomal protein, large, P1	P47955	147757.781	0.000518	upregulation
NKST(ph)ESLQANVQR	ribosomal protein L13	P47963	142350.844	0.0204	upregulation
GRASS(ph)HSSQSQGGGSVTK	lamin A/C	P48678	7.028	0.027	upregulation
LDNARQS(ph)AER	lamin A/C	P48678	7.049	0.00922	upregulation
SES(ph)PKEPEQLRK	heterogeneous nuclear ribonucleoprotein A1	P49312	5.473	0.0158	upregulation
YVDSEGHLYT(ph)VPIR	caveolin 1	P49817	3.55	0.0434	upregulation
STYQDKPST(ph)PAEKK	calpastatin	P51125	3.545	0.0482	upregulation
KQQQEPTCEPS(ph)PK	high mobility group AT-hook 2	P52927	5.393	0.0165	upregulation
SRSLS(ph)ASPALGSTK	NAD kinase	P58058	11.513	0.00144	upregulation
VSGAGLS(ph)PSRK	tankyrase 1 binding protein 1	P58871	429301.688	0.0137	upregulation
DVGHLEEGASGGLLS(ph)PSTPHSR	tankyrase 1 binding protein 1	P58871	431456.812	0.0042	upregulation
S(ph)PVGDTGLGKR	tankyrase 1 binding protein 1	P58871	440060.156	0.00247	upregulation
S(ph)HTGEAAAVR	BCL2 like 13	P59017	2.946	0.013	upregulation

LKAGGS(ph)VESLR	SAM and SH3 domain containing 1	P59808	179679.312	0.000742	upregulation
S(ph)LTEGEMKK	SAM and SH3 domain containing 1	P59808	181837.375	0.0324	upregulation
VISS(ph)IEQK	tyrosine 3-monooxygenase/tryptophan 5-monooxygenase activation protein gamma	P61982	887101	0.00174	upregulation
VISSIEQKTS(ph)ADGNEK	tyrosine 3-monooxygenase/tryptophan 5-monooxygenase activation protein gamma	P61982	900244.438	0.0152	upregulation
LSS(ph)LRAS(ph)TSKSESSQK	ribosomal protein S6	P62754	162951.625	0.0224	upregulation
LSS(ph)LRASTSK	ribosomal protein S6	P62754	164898.359	0.000941	upregulation
NYQQNYQNSESGEKNEGSES(ph)APEGQAQQR	Y-box binding protein 1	P62960	780434.062	0.00000113	upregulation
NYQQNYQNSES(ph)GEKNEGSESAPEGQAQQR	Y-box binding protein 1	P62960	795309.625	0.0448	upregulation
RSPS(ph)PYYSR	transformer 2 beta homolog	P62996	507444.219	0.0000693	upregulation
TAS(ph)EGDGGAAGGAGTAGGRPMSVAGSPLS(ph)PGPVR	insulin receptor substrate 2	P81122	6.153	0.0283	upregulation
TAS(ph)EGDGGAAGGAGTAGGRPMSVAGSPLS(ph)PGPVR	insulin receptor substrate 2	P81122	6.198	0.0159	upregulation
TAS(ph)EGDGGAAGGAGTAGGRPMSVAGS(ph)PLSPGPVR	insulin receptor substrate 2	P81122	6.217	0.0102	upregulation
KADS(ph)DSEDKGEESKPK	chromobox 1	P83917	3.555	0.0448	upregulation
LMELHGEGGSS(ph)GK	ribosomal protein S3A1	P97351	154308.781	0.0191	upregulation
FLTQS(ph)PK	lysosomal trafficking regulator	P97412	7.308	0.0172	upregulation
STANHDSES(ph)PVHSPSAHR	lysosomal trafficking regulator	P97412	7.33	0.0325	upregulation
MDIDRS(ph)PGLLGTPDLK	myosin phosphatase Rho interacting protein	P97434	9.859	0.021	upregulation
TTSRS(ph)PVLSR	mitogen-activated protein kinase kinase kinase kinase 4	P97820	7.974	0.0056	upregulation
GSSNKDFT(ph)PGRDK	RB binding protein 6, ubiquitin ligase	P97868	119664.195	0.000155	upregulation
SRS(ph)PQAFR	RB binding protein 6, ubiquitin ligase	P97868	125077.453	0.0022	upregulation
LERT(ph)PEKDK	RB binding protein 6, ubiquitin ligase	P97868	125606.266	0.02	upregulation
IHVS(ph)DQELQSANASVDDSRLEELK	ubiquitin like modifier activating enzyme 1	Q02053	546198.938	0.000417	upregulation
SRNS(ph)PLLDR	microtubule affinity regulating kinase 2	Q05512	8.249	0.00425	upregulation
TAS(ph)EGS(ph)EAETPEAPKQPAKK	La ribonucleoprotein domain family member 7	Q05CL8	6.721	0.0493	upregulation

TAS(ph)EGSEAETPEAPKQPAK	La ribonucleoprotein domain family member 7	Q05CL8	6.785	0.0208	upregulation
TARPNSEAPLS(ph)GSEDADDSNKLSKK	eukaryotic translation initiation factor 5B	Q05D44	4.538	0.000238	upregulation
TARPNSEAPLS(ph)GSEDADDSNKLSK	eukaryotic translation initiation factor 5B	Q05D44	4.54	0.0206	upregulation
MGS(ph)PKPER	zinc finger CCCH-type containing 18	Q0P678	1091479.875	0.00782	upregulation
VQSQEETRS(ph)DEEDRASEPK	zinc finger CCCH-type containing 18	Q0P678	1207649.5	0.00229	upregulation
KKLGVSVS(ph)PSR	zinc finger CCCH-type containing 18	Q0P678	1254159.5	0.0367	upregulation
GGS(ph)REYETGGGSSSSR	RNA binding motif protein 15	Q0VBL3	128906.766	0.00446	upregulation
RGS(ph)PVPPVPERR	family with sequence similarity 83 member H	Q148V8	4.935	0.0188	upregulation
KGSPT(ph)PAYPER	family with sequence similarity 83 member H	Q148V8	4.958	0.0168	upregulation
EKEPEAASSRGS(ph)PVR	ubiquitin specific peptidase 39	Q3TIX9	607535.812	0.00308	upregulation
VGDTEKPEPERS(ph)PPNR	chromosome 9 open reading frame 78	Q3TQI7	3.367	0.0202	upregulation
KAKPAMPQDSVPS(ph)PR	ATP citrate lyase	Q3TS02	1.464	0.0118	upregulation
KSYESSEDCPEAASS(ph)PTRK	alkB homolog 5, RNA demethylase	Q3TSG4	2.192	0.0431	upregulation
GS(ph)PQQIDHAK	KH-type splicing regulatory protein	Q3U0V1	6.396	0.0136	upregulation
HGSGPNIILTGDSS(ph)PGFSK	CREB regulated transcription coactivator 2	Q3U182	3.941	0.0208	upregulation
SPS(ph)APVCK	family with sequence similarity 117 member B	Q3U3E2	4.732	0.0273	upregulation
KSRS(ph)DNALNLVTER	prickle planar cell polarity protein 1	Q3U5C7	69.078	0.0000562	upregulation
SDCQRS(ph)PPGVLK	TRAF-type zinc finger domain containing 1	Q3UDK1	512177.938	0.00135	upregulation
STS(ph)VDDTDKSSSEAIMVR	cordon-bleu WH2 repeat protein like 1	Q3UMF0	3.886	0.0314	upregulation
KREQT(ph)ASAPATPLVSK	cordon-bleu WH2 repeat protein like 1	Q3UMF0	3.907	0.0414	upregulation
QDS(ph)NPKPKPSNEITR	cordon-bleu WH2 repeat protein like 1	Q3UMF0	3.919	0.0394	upregulation
LPEKEPACTYGNNVPLS(ph)PVDGSNKNPAASYLK	cordon-bleu WH2 repeat protein like 1	Q3UMF0	3.926	0.044	upregulation
TRLAS(ph)ESANDDNEDS	HDGF like 2	Q3UMU9	5.275	0.00228	upregulation
S(ph)EGLSLER	HDGF like 2	Q3UMU9	5.297	0.0255	upregulation
AQEDGQDS(ph)EDGPRGGSSEELHDSPR	HDGF like 2	Q3UMU9	5.314	0.0367	upregulation
LAS(ph)ESANDDNEDS	HDGF like 2	Q3UMU9	5.376	0.0174	upregulation
HTAPSS(ph)PSPGTR	transforming growth factor beta receptor associated protein 1	Q3UR70	398095.906	0.00148	upregulation
MSGS(ph)PGVSR	splicing factor SWAP	Q3USH5	200686.609	0.00142	upregulation

S(ph)NSLPHSAVSNAASK	WD repeat domain 20	Q3UWE6	649360.25	0.000236	upregulation
VPS(ph)PDHR	pre-mRNA processing factor 38A	Q4FK66	112.388	0.000388	upregulation
QS(ph)PSPSTRPIR	serine/arginine repetitive matrix 1	Q52KI8	251986.859	0.000305	upregulation
TRHS(ph)PTPQQSNR	serine/arginine repetitive matrix 1	Q52KI8	253254.438	0.000809	upregulation
SS(ph)SKDSRPSQAAGDNQGDEAKEQTFSGGTSQDIK	thyroid hormone receptor associated protein 3	Q569Z6	404227.031	0.0209	upregulation
ERS(ph)PALK	thyroid hormone receptor associated protein 3	Q569Z6	408076.062	0.00845	upregulation
NKKS(ph)PEIHR	thyroid hormone receptor associated protein 3	Q569Z6	410903.125	0.0000188	upregulation
RSSMSSCGSSGYFSSSPT(ph)LS(ph)SSPPVLCNPK	DEP domain containing MTOR interacting protein	Q570Y9	4.198	0.043	upregulation
GPRPCTS(ph)PQPLR	PEAK1 related, kinase-activating pseudokinase 1	Q571I4	62.941	0.000701	upregulation
SDDSKSSS(ph)PEPVTHLK	Rho GTPase activating protein 1	Q5FWK3	2.457	0.0179	upregulation
VHAS(ph)RPASLDSGR	coiled-coil domain containing 88A	Q5SNZ0	3.654	0.0417	upregulation
ESDTKNEVNGTSEDIKSEGDTQS(ph)N	LUC7 like 3 pre-mRNA splicing factor	Q5SUF2	7.295	0.00601	upregulation
LNS(ph)DEEGESSGKR	LEO1 homolog, Paf1/RNA polymerase II complex component	Q5XJE5	6.86	0.017	upregulation
LNS(ph)DEEGESSGK	LEO1 homolog, Paf1/RNA polymerase II complex component	Q5XJE5	6.907	0.00903	upregulation
KLNS(ph)DEEGESSGKR	LEO1 homolog, Paf1/RNA polymerase II complex component	Q5XJE5	6.929	0.0301	upregulation
SAEPPRS(ph)PLLKR	microtubule associated serine/threonine kinase 2	Q60592	8.654	0.0153	upregulation
RES(ph)PPIR	nuclear receptor corepressor 1	Q60974	11.581	0.0181	upregulation
GGT(ph)YPRR	mitogen-activated protein kinase kinase kinase 3	Q61084	7.827	0.0281	upregulation
TLS(ph)PGRR	pre-mRNA processing factor 4B	Q61136	197.745	0.00000147	upregulation
IGS(ph)DPLAYEPK	solute carrier family 9 member A1	Q61165	233702.266	0.0000531	upregulation
YSDEQLNSGRQS(ph)PSQNER	APC, WNT signaling pathway regulator	Q61315	2.293	0.0489	upregulation
HAEQNGPVDGQGDNPGSQAAEHGADT(ph)AVPSDGDK	heat shock protein family A (Hsp70) member 4	Q61316	5.777	0.0455	upregulation
SS(ph)FSNSADDIK	chromobox 5	Q61686	3.594	0.0487	upregulation
KSS(ph)FSNSADDIKSK	chromobox 5	Q61686	3.598	0.00688	upregulation
SEMKTELS(ph)PRPGAAGR	eukaryotic translation initiation	Q61749	4.351	0.0469	upregulation

	factor 2B subunit delta				
DLKPST(ph)PR	nucleophosmin 1	Q61937	12.83	0.021	upregulation
LHQLAMQQSHFPMTHGNTGFSGIESSS(ph)PEVK	poly(rC) binding protein 2	Q61990	14.692	0.00563	upregulation
LHQLAMQQSHFPMTHGNTGFSGIESSS(ph)PEVK	poly(rC) binding protein 2	Q61990	15.999	0.00139	upregulation
TS(ph)PDTLR	serine and arginine rich splicing factor 2	Q62093	345134.5	0.0125	upregulation
TS(ph)PDTLRR	serine and arginine rich splicing factor 2	Q62093	353324.062	0.0098	upregulation
LRS(ph)PFLQK	drebrin like	Q62418	4.085	0.0378	upregulation
QLT(ph)QPETSYGR	drebrin like	Q62418	4.119	0.0322	upregulation
SVS(ph)VDSGEQR	B cell CLL/lymphoma 9 like	Q67FY2	3.036	0.0353	upregulation
HTSS(ph)PEVVAEDR	RHO family interacting cell polarization regulator 1	Q68FE6	141968.828	0.00105	upregulation
LGS(ph)LSAR	centrosomal protein 170	Q6A065	3.852	0.0311	upregulation
ADTS(ph)PTYVR	zinc finger protein 219	Q6IQX8	1488215.875	0.0204	upregulation
KLS(ph)VGGDSDPPLKR	zinc finger CCCH-type containing 11A	Q6NZF1	906670.438	0.00747	upregulation
RSS(ph)PLLR	histone deacetylase 4	Q6NZM9	5.211	0.0284	upregulation
RLNHS(ph)PPQSSSR	RNA binding motif protein 26	Q6NZN0	130183.766	0.0162	upregulation
EAEQGS(ph)GEEKEEKEGDLK	ATP binding cassette subfamily F member 1	Q6P542	-7.89	0.025	downregulation
AAIS(ph)QLR	oxidative stress responsive 1	Q6P9R2	13.994	0.0419	upregulation
EQGVESPGAQPASS(ph)PR	taxilin alpha	Q6PAM1	537618.75	0.00233	upregulation
ERSMS(ph)ENAVR	mitochondrial fission factor	Q6PCP5	9.048	0.0125	upregulation
TLTDEVNS(ph)PDSDRR	SWI/SNF related, matrix associated, actin dependent regulator of chromatin subfamily c member 2	Q6PDG5	238068.422	0.00747	upregulation
EVAES(ph)PRPR	pleckstrin homology like domain family B member 1	Q6PDH0	18.817	0.000275	upregulation
ESTESSNTT(ph)IEDEDVKAR	calcium/calmodulin dependent protein kinase II delta	Q6PHZ2	3.453	0.042	upregulation
ESTESSNTT(ph)IEDEDVK	calcium/calmodulin dependent protein kinase II delta	Q6PHZ2	3.469	0.0412	upregulation
KDS(ph)QNSSQHSVSSHR	membrane associated guanylate kinase, WW and PDZ domain containing 1	Q6RHR9	7.622	0.0476	upregulation
KDSQNSS(ph)QHSVSSHR	membrane associated guanylate kinase, WW and PDZ domain containing 1	Q6RHR9	7.675	0.0061	upregulation

GVAPADS(ph)PDAPRR	ankyrin repeat and IBR domain containing 1	Q6ZPS6	2.26	0.0282	upregulation
TGTGSPFAGNS(ph)PAR	zinc finger CCCH-type containing 4	Q6ZPZ3	1313716.75	0.0319	upregulation
S(ph)LPTTVPES(ph)PNYR	La ribonucleoprotein domain family member 1	Q6ZQ58	6.401	0.00651	upregulation
S(ph)LPTTVPESPNY(ph)R	La ribonucleoprotein domain family member 1	Q6ZQ58	6.544	0.0189	upregulation
SSPSTGS(ph)LDSGNESKEK	ArfGAP with coiled-coil, ankyrin repeat and PH domains 2	Q6ZQK5	1.213	0.0182	upregulation
AEVSTIHLQS(ph)PGRK	synemin	Q70IV5	361914.219	0.000992	upregulation
SRS(ph)LGGAVGSASGGR	zinc and ring finger 2	Q71FD5	1701507.625	0.0437	upregulation
ERSDS(ph)GGSSSEPFER	proline rich coiled-coil 2A	Q7TSC1	84038.047	0.0359	upregulation
SDS(ph)GGSSSEPFER	proline rich coiled-coil 2A	Q7TSC1	87469.734	0.00729	upregulation
AAAS(ph)PLGESGPSIHPHDK	CTD phosphatase subunit 1	Q7TSG2	3.949	0.0415	upregulation
S(ph)KSEDMDSVESK	activating transcription factor 7 interacting protein	Q7TT18	2.679	0.0453	upregulation
RIS(ph)QSQPVR	ubiquitin protein ligase E3 component n-recognin 5	Q80TP3	569948.875	0.00532	upregulation
VAS(ph)DTEDTDRITSK	arginine-glutamic acid dipeptide repeats	Q80TZ9	138723.625	0.0446	upregulation
EKVAS(ph)DTEDTDRITSK	arginine-glutamic acid dipeptide repeats	Q80TZ9	139093.719	0.0239	upregulation
GKAS(ph)PFEEDQNR	pumilio RNA binding family member 2	Q80U58	94433.523	0.0433	upregulation
SHS(ph)DTNIASR	RUN and cysteine rich domain containing beclin 1 interacting protein	Q80U62	173510.859	0.0311	upregulation
RSSFS(ph)EGQTAPVASGTKK	RUN and cysteine rich domain containing beclin 1 interacting protein	Q80U62	173548.797	0.0168	upregulation
RSS(ph)FSEGQTAPVASGTKK	RUN and cysteine rich domain containing beclin 1 interacting protein	Q80U62	176012.656	0.00725	upregulation
SRS(ph)PIER	vestigial like family member 4	Q80V24	622769.062	0.0295	upregulation
SANDSTVHS(ph)PFTKR	ubiquitin-associated protein 2-like	Q80X50	554778.188	0.000276	upregulation
AHTDEPHS(ph)PQSK	elaC ribonuclease Z 2	Q80Y81	4.607	0.0229	upregulation
RAHTDEPHS(ph)PQSK	elaC ribonuclease Z 2	Q80Y81	4.632	0.00514	upregulation
TAS(ph)NAEQYK	actin filament associated protein 1	Q80YS6	1.805	0.0213	upregulation
SMGTGDSAGVEVPSS(ph)PLRR	zinc finger C3HC-type containing 1	Q80YV2	1357371.375	0.00204	upregulation
LCS(ph)SSSSDTSPR	zinc finger C3HC-type containing 1	Q80YV2	1453507.75	0.000000962	upregulation

SQS(ph)LPGADSLLAKPIDK	transformation/transcription domain associated protein	Q80YV3	521163.812	0.00243	upregulation
EQMMNSSVSSGSGS(ph)LR	discs large MAGUK scaffold protein 1	Q811D0	4.216	0.0372	upregulation
SS(ph)PTPHSGPCPSR	proline rich 5	Q812A5	365.096	0.00368	upregulation
AEEGGESEGDAS(ph)EKDAK	HIV-1 Tat specific factor 1	Q8BGC0	5.834	0.0482	upregulation
SRTGSES(ph)SQTGASATSGR	eukaryotic translation initiation factor 4B	Q8BGD9	4.472	0.00825	upregulation
TGS(ph)ESSQTGASATSGR	eukaryotic translation initiation factor 4B	Q8BGD9	4.507	0.0351	upregulation
TGSESS(ph)QTGASATSGR	eukaryotic translation initiation factor 4B	Q8BGD9	4.511	0.0232	upregulation
LIHQFLDEDSDPMLS(ph)PR	family with sequence similarity 13, member A	Q8BGI4	4.872	0.0403	upregulation
SRS(ph)QPCVLNDK	family with sequence similarity 53 member B	Q8BGR5	4.904	0.00402	upregulation
FGGQPCQGAPGSAPCGQAGDSWS(ph)PDPHPVGGGR	family with sequence similarity 53 member B	Q8BGR5	4.917	0.0249	upregulation
SRS(ph)ESETSTMAAK	chromosome 18 open reading frame 25	Q8BH50	3.342	0.0308	upregulation
HGAPAAPS(ph)PPPR	TBC1 domain family member 10B	Q8BHL3	373326.906	0.0232	upregulation
VVPQQITHTS(ph)PR	Sin3A associated protein 130	Q8BIH0	179527.391	0.00167	upregulation
LS(ph)PEPVAHR	Smad nuclear interacting protein 1	Q8BIZ6	243366.234	0.0244	upregulation
SRT(ph)PSASHEEQQE	small glutamine rich tetratricopeptide repeat containing alpha	Q8BJU0	202985.25	0.00525	upregulation
TPS(ph)ASHEEQQE	small glutamine rich tetratricopeptide repeat containing alpha	Q8BJU0	206196.203	0.000175	upregulation
SRTPSAS(ph)HEEQQE	small glutamine rich tetratricopeptide repeat containing alpha	Q8BJU0	207915.266	0.000201	upregulation
VENDENNETLS(ph)EPGESSKEENCSK	SWI5 dependent homologous recombination repair protein 1	Q8BP27	200505.812	0.0187	upregulation
SSPGNLRDQS(ph)PK	protein phosphatase 1 regulatory subunit 18	Q8BQ30	52.108	0.000722	upregulation
LAGSGDDS(ph)PKRK	protein phosphatase 1 regulatory subunit 18	Q8BQ30	55.956	0.0427	upregulation
LKSGMS(ph)PEQSK	serine/arginine repetitive matrix 2	Q8BTI8	277516	0.00431	upregulation
HSLSGSS(ph)PGMK	serine/arginine repetitive matrix 2	Q8BTI8	284995.781	0.00194	upregulation
QSSS(ph)PYEDKDKK	serine/arginine repetitive matrix 2	Q8BTI8	294071.25	0.00559	upregulation
REISSS(ph)PTSK	serine/arginine repetitive matrix 2	Q8BTI8	295464.75	0.00998	upregulation

QSHSESS(ph)PDGEVK	serine/arginine repetitive matrix 2	Q8BTI8	295539.656	0.000127	upregulation
SRTS(ph)PVTR	serine/arginine repetitive matrix 2	Q8BTI8	298254.969	0.0015	upregulation
DGLPRT(ph)PSRR	serine/arginine repetitive matrix 2	Q8BTI8	300038.656	0.00156	upregulation
SGS(ph)SQELDGKPSASPQER	serine/arginine repetitive matrix 2	Q8BTI8	302599.844	0.0000597	upregulation
SGSS(ph)QELDGKPSASPQER	serine/arginine repetitive matrix 2	Q8BTI8	305640.469	0.00658	upregulation
SRTS(ph)PVSR	serine/arginine repetitive matrix 2	Q8BTI8	315176.75	0.00495	upregulation
SVT(ph)PQRER	serine/arginine repetitive matrix 2	Q8BTI8	317213.5	0.000398	upregulation
SRS(ph)GSSQELDGKPSASPQER	serine/arginine repetitive matrix 2	Q8BTI8	333835.469	0.0296	upregulation
HSLS(ph)GSSPGMKDTPQTPSR	serine/arginine repetitive matrix 2	Q8BTI8	333853.594	0.000171	upregulation
SST(ph)PSSPTGTSSTDSGGQHLGWGEQQGQWLR	phosphorylase kinase regulatory subunit alpha 2	Q8BWJ3	18.591	0.00888	upregulation
NGKEQYVPPRS(ph)PK	La ribonucleoprotein domain family member 4	Q8BWW4	6.553	0.0274	upregulation
SRS(ph)QPCDLDAR	family with sequence similarity 53 member C	Q8BXQ8	4.923	0.0244	upregulation
LGS(ph)MDSFER	TBC1 domain family member 4	Q8BYJ6	374882.25	0.00000503	upregulation
HAS(ph)APSHVQPSDSEK	TBC1 domain family member 4	Q8BYJ6	380509.719	0.000613	upregulation
LGS(ph)MDSFER	TBC1 domain family member 4	Q8BYJ6	380811.594	3.24E-08	upregulation
ST(ph)TPVKEKEHSK	splicing regulatory glutamic acid and lysine rich protein 1	Q8BZX4	243549.375	0.0145	upregulation
AADEKGS(ph)PRTEDEGK	splicing regulatory glutamic acid and lysine rich protein 1	Q8BZX4	245942.422	0.0117	upregulation
KAPARPSS(ph)ASATPR	microtubule associated protein 1S	Q8C052	7.758	0.00382	upregulation
FNSDSHS(ph)PK	Rho GTPase activating protein 12	Q8C0D4	2.497	0.0412	upregulation
SQCRNSPS(ph)NLSSSSETGSGGGTYR	signal induced proliferation associated 1 like 1	Q8C0T5	223966.734	0.0303	upregulation
EYGST(ph)SSIDK	signal induced proliferation associated 1 like 1	Q8C0T5	226211.891	0.00000849	upregulation
LSS(ph)TGGQTPR	IWS1, SUPT6H interacting protein	Q8C1D8	6.377	0.00956	upregulation
S(ph)PTKSSLDYR	RNA binding motif protein 14	Q8C2Q3	127297.102	0.00641	upregulation
SPLRRS(ph)PPR	RNA binding motif protein 14	Q8C2Q3	128333.398	0.00242	upregulation
TNSMSSSGLGS(ph)PNR	CCR4-NOT transcription complex subunit 2	Q8C5L3	3.875	0.0251	upregulation
YTEQERS(ph)PR	CWC22 spliceosome associated protein homolog	Q8C5N3	4.016	0.0307	upregulation
TAS(ph)LNQRPR	abl interactor 1	Q8CBW3	-2.943	0.0366	downregulation
LGSQHS(ph)PGR	abl interactor 1	Q8CBW3	-2.294	0.0489	downregulation

HNS(ph)TTSSTSSGGYR R	abl interactor 1	Q8CBW3	1	0.000492	upregulation
LLLLASS(ph)PTER	Rho GTPase activating protein 29	Q8CGF1	2.612	0.0455	upregulation
GRRPS(ph)GPEGAAR	polyhomeotic homolog 3	Q8CHP6	16.66	0.000185	upregulation
ISQRDPS(ph)PESK	polyhomeotic homolog 3	Q8CHP6	17.232	0.00166	upregulation
KGGS(ph)PGLESR	polyhomeotic homolog 3	Q8CHP6	17.601	0.0174	upregulation
SPS(ph)QEPSAPGKAEA VGEQAR	eukaryotic translation initiation factor 3 subunit B	Q8JZQ9	4.36	0.0466	upregulation
KAEGEPQEES(ph)PLKS K	BCL2 associated transcription factor 1	Q8K019	3.062	0.00836	upregulation
LRCDS(ph)ADLR	BCL2 associated transcription factor 1	Q8K019	3.067	0.0293	upregulation
EVQS(ph)PEQVKSEK	BCL2 associated transcription factor 1	Q8K019	3.076	0.00772	upregulation
EVQS(ph)PEQVK	BCL2 associated transcription factor 1	Q8K019	3.093	0.0461	upregulation
QKS(ph)PEIHR	BCL2 associated transcription factor 1	Q8K019	3.118	0.0369	upregulation
S(ph)PAKTITPQNAPR	BCL2 associated transcription factor 1	Q8K019	3.153	0.0325	upregulation
KAEGEPQEES(ph)PLK	BCL2 associated transcription factor 1	Q8K019	3.162	0.0295	upregulation
RYSSSGT(ph)PSSASPAL SR	nuclear factor of activated T cells 4	Q8K120	12.346	0.0186	upregulation
ELPSAGS(ph)RDK	dynamin 1 like	Q8K1M6	4.233	0.0241	upregulation
STQSENQHQGAQDTSD LMS(ph)PSKR	integrator complex subunit 10	Q8K2A7	5.95	0.0222	upregulation
_EKTMSS(ph)DDEECSAK _	integrator complex subunit 10	Q8K2A7	5.962	0.0157	upregulation
SYS(ph)PDGKESPSDK	matrin 3	Q8K310	8.859	0.0235	upregulation
SYS(ph)PDGKESPSDKK	matrin 3	Q8K310	9.029	0.00283	upregulation
GT(ph)VTGERQSGDGQ ESTEPVENKVGK	cancer susceptibility 3	Q8K3W3	3.479	0.0483	upregulation
GTVTGERQS(ph)GDGQ ESTEPVENK	cancer susceptibility 3	Q8K3W3	3.488	0.0465	upregulation
DAPRPDHPPHDGHS(p h)PASR	WD repeat domain 33	Q8K4P0	668138.5	0.00392	upregulation
HDLDAS(ph)PPR	BUD13 homolog	Q8R149	3.307	0.0315	upregulation
HDLDAS(ph)PPRK	BUD13 homolog	Q8R149	3.327	0.0394	upregulation
S(ph)MSIDDTPR	dedicator of cytokinesis 7	Q8R1A4	4.251	0.0252	upregulation
KPASVSPTTPTS(ph)PTE GEAS	dynein cytoplasmic 1 light intermediate chain 1	Q8R1Q8	4.275	0.0252	upregulation
SS(ph)PVHIIATSK	HMG-box transcription factor 1	Q8R316	5.211	0.0234	upregulation
S(ph)PGPPALKHPTSK	interferon regulatory factor 2 binding protein 1	Q8R3Y8	5.983	0.00873	upregulation
_RKAS(ph)PEPEGETAGK _	interferon regulatory factor 2 binding protein 1	Q8R3Y8	5.994	0.0186	upregulation
VEEEQEADEEDVS(ph)E EEAEDREGASK	thioredoxin related transmembrane protein 1	Q8VBT0	425174.281	0.0000269	upregulation
GTGDCS(ph)DEEVDGK ADGADAK	myosin heavy chain 9	Q8VDD5	11.248	0.0242	upregulation

KRVPS(ph)PLPK	UBA domain containing 1	Q8VDI7	550638.062	0.00227	upregulation
RPSS(ph)GGEEEEKAR	cell cycle and apoptosis regulator 2	Q8VDP4	3.599	0.0174	upregulation
YRS(ph)PYSGPK	RNA binding motif protein 39	Q8VH51	131062.242	0.0138	upregulation
LGS(ph)FGSITR	filamin C	Q8VHX6	5.002	0.00529	upregulation
KAS(ph)LTHEQQQSAR	coiled-coil-helix-coiled-coil-helix domain containing 6	Q91VN4	3.854	0.0346	upregulation
TSSFT(ph)DQLDDVTPNR	golgin A4	Q91VW5	5.159	0.015	upregulation
KVDSPFSSGS(ph)PSR	protein kinase AMP-activated non-catalytic subunit gamma 2	Q91WG5	82.043	0.0000292	upregulation
IYASSS(ph)PPDTGQR	protein kinase AMP-activated non-catalytic subunit gamma 2	Q91WG5	84.075	0.00526	upregulation
RPAAAAAAGSAS(ph)PR	Werner helicase interacting protein 1	Q91XU0	718199.312	0.000541	upregulation
AQSNGSGNGSDS(ph)EMDTSSLER	Rho GTPase activating protein 35	Q91YM2	2.618	0.0186	upregulation
EPLPPFENQDMHSAS(ph)A	lipin 1	Q91ZP3	7.056	0.00506	upregulation
SS(ph)PPAT(ph)DPGPVPSSPSQEPPTKR	UBX domain protein 1	Q922Y1	577531.812	0.000457	upregulation
CGETVES(ph)GDEKDLAK	debranching RNA lariats 1	Q923B1	4.125	0.0275	upregulation
S(ph)EDDSAVPVAK	WW domain binding protein 11	Q923D5	627204.062	0.0121	upregulation
NSSQTGGKPGSS(ph)PITK	mediator complex subunit 1	Q925J9	9.032	0.0102	upregulation
ETNVSKEDTDQEEKAS(ph)NEDVTK	PC4 and SFRS1 interacting protein 1	Q99JF8	87906.289	0.0191	upregulation
ALAHNGT(ph)PR	Ras related GTP binding C	Q99K70	165032.312	0.00401	upregulation
ALAHNGT(ph)PR	Ras related GTP binding C	Q99K70	165049.484	0.0023	upregulation
HELS(ph)PPQKR	serrate, RNA effector molecule	Q99MR6	334087.906	0.0334	upregulation
AAHTEDINACTLTTS(ph)PR	Raf-1 proto-oncogene, serine/threonine kinase	Q99N57	116386.32	0.0446	upregulation
TT(ph)PAPSPGSANESFFAPSR	MID1 interacting protein 1	Q9CQ20	9.345	0.00321	upregulation
PGPTPSGTNVGSSGRS(ph)PSK	Sec61 translocon beta subunit	Q9CQS8	195449.5	0.0000429	upregulation
PGPTPSGTNVGS(ph)SGRSPSK	Sec61 translocon beta subunit	Q9CQS8	196025.062	0.00227	upregulation
TRTFS(ph)ATVR	calcium regulated heat stable protein 1	Q9CR86	3.479	0.0496	upregulation
AAGGGGGS(ph)GEDEAQSRR	golgi phosphoprotein 3	Q9CRA5	5.197	0.0237	upregulation
TSS(ph)LTHSEEK	RAN binding protein 3	Q9CT10	117715.508	0.000000636	upregulation
KGDVEGSQS(ph)QDEGEGSGESER	structural maintenance of chromosomes 3	Q9CW03	242414.312	0.0229	upregulation
HKMS(ph)PPPSSFNEPR	ribonucleoprotein, PTB binding 1	Q9CW46	117864.594	0.0162	upregulation

GTGRPNS(ph)PQRLDR	CD2 cytoplasmic tail binding protein 2	Q9CWK3	3.684	0.0379	upregulation
LGSTAPQVLNTSS(ph)PAQQAENEAK	NSFL1 cofactor	Q9CZ44	12.976	0.000192	upregulation
MGLS(ph)MDR	heterogeneous nuclear ribonucleoprotein M	Q9D0E1	5.625	0.0119	upregulation
MGSS(ph)IER	heterogeneous nuclear ribonucleoprotein M	Q9D0E1	5.653	0.0396	upregulation
MGLS(ph)MDR	heterogeneous nuclear ribonucleoprotein M	Q9D0E1	5.675	0.0441	upregulation
RTS(ph)AETADVATSELLVNQASTNPVPGDGLHR	target of EGR1, exonuclease	Q9D2E2	488856.906	0.00209	upregulation
DTTISNHS(ph)R	angiomotin like 1	Q9D4H4	2.229	0.0224	upregulation
KFS(ph)EEPEVAANFTK	NOP56 ribonucleoprotein	Q9D6Z1	12.56	0.00228	upregulation
RNS(ph)TTFPSR	family with sequence similarity 122A	Q9DB52	4.784	0.0188	upregulation
RNST(ph)TFPSR	family with sequence similarity 122A	Q9DB52	4.851	0.0365	upregulation
NS(ph)PGCQVASNPR	5'-3' exoribonuclease 2	Q9DBR1	766115.688	0.0311	upregulation
LAST(ph)SDIEEKENR	protein phosphatase 1 regulatory subunit 12A	Q9DBR7	36.277	0.0186	upregulation
LAS(ph)TSDIEEKENR	protein phosphatase 1 regulatory subunit 12A	Q9DBR7	37.307	0.00148	upregulation
LSSS(ph)LDNKEKEK	protein phosphatase 1 regulatory subunit 12A	Q9DBR7	46.01	0.00119	upregulation
NGS(ph)PPPGAPASR	TSC22 domain family member 4	Q9EQN3	526604.875	0.000878	upregulation
RPAATDS(ph)PKLSAK	MLLT1, super elongation complex subunit	Q9ERL0	9.561	0.0396	upregulation
VSGRRS(ph)PEPCSKPEK	MLLT1, super elongation complex subunit	Q9ERL0	9.569	0.0258	upregulation
SQSSEGVSSLSS(ph)S(ph)PSNSLETQSQSLSR	ubiquitination factor E4B	Q9ES00	562159.938	0.00291	upregulation
SSS(ph)PVGLAK	eukaryotic translation initiation factor 4E nuclear import factor 1	Q9EST3	4.532	0.0127	upregulation
VKVVEEMS(ph)E	dyskerin pseudouridine synthase 1	Q9ESX5	4.208	0.0317	upregulation
NRDGNPGGPGSERGPS(ph)V	tumor suppressing subtransferable candidate 4	Q9JHE7	532295.5	0.000468	upregulation
TDS(ph)TSDGRPAWMR	dynein cytoplasmic 1 heavy chain 1	Q9JHU4	4.255	0.0101	upregulation
RSS(ph)PEPDGGATPK	SH2B adaptor protein 2	Q9JID9	209256.594	0.00291	upregulation
S(ph)SEDVSAHAATK	SH2B adaptor protein 2	Q9JID9	211085.703	0.0132	upregulation
SS(ph)EDVSAHAATK	SH2B adaptor protein 2	Q9JID9	217370.266	0.00424	upregulation

SSSFSEEKGES(ph)DDEKPR	apoptotic chromatin condensation inducer 1	Q9JIX8	1.254	0.0422	upregulation
ISEDET(ph)ERNGDDGTHDK	apoptotic chromatin condensation inducer 1	Q9JIX8	1.464	0.0197	upregulation
S(ph)PAASGAPQAPAPAALLAGSPGGDAAPGPAPASSAPAGGEDAEKK	Y-box binding protein 3	Q9JKB3	795532.375	0.0115	upregulation
EKREEEEDNEDDGS(ph)DLGEALA	chromatin accessibility complex 1	Q9JKP8	3.858	0.0348	upregulation
S(ph)PGAVYPK	CD2 associated protein	Q9JLQ0	3.683	0.0433	upregulation
LEKQNSTPESDY(ph)DNTACDPEPDDTGSTR	GIT ArfGAP 2	Q9JLQ2	5.053	0.00681	upregulation
EADS(ph)KPVSQKSPPPAEKVEVK	BCL2 associated athanogene 3	Q9JLV1	2.921	0.0258	upregulation
VDDKPSS(ph)PGDSSKK	chromobox 8	Q9QXV1	3.598	0.0423	upregulation
VNVPEESRNGETS(ph)PR	adducin 3	Q9QYB5	1.786	0.0304	upregulation
QKGS(ph)EENLDETR	adducin 1	Q9QYC0	1.517	0.0196	upregulation
SPT(ph)PGKGPVDR	microtubule associated protein 1A	Q9QYR6	7.71	0.0409	upregulation
SRPS(ph)SPAVR	zinc finger, RAN-binding domain containing 2	Q9R020	1880025.375	0.0000479	upregulation
YNLDAS(ph)EEEDSNKK	zinc finger, RAN-binding domain containing 2	Q9R020	2125133.75	0.0055	upregulation
EVEDKES(ph)EGEEEDEDEDLSK	zinc finger, RAN-binding domain containing 2	Q9R020	2586337	0.011	upregulation
SRPSS(ph)PAVR	zinc finger, RAN-binding domain containing 2	Q9R020	4058515.75	0.000353	upregulation
SRPSS(ph)PAVRK	zinc finger, RAN-binding domain containing 2	Q9R020	7840429.5	0.00037	upregulation
QDPIPGS(ph)PDNSR	DLC1 Rho GTPase activating protein	Q9R0Z9	4.21	0.0276	upregulation
SESKHKS(ph)PK	pre-mRNA processing factor 40 homolog A	Q9R1C7	139.465	0.01	upregulation
HRAEAPPLQREDSGT(ph)FSLGK	protein activator of interferon induced protein kinase EIF2AK2	Q9WTX2	84.479	0.00364	upregulation
KGAEEEEEEEDDDS(ph)EEEIK	dematin actin binding protein	Q9WV69	4.228	0.0438	upregulation
TS(ph)PEPQRENASPAPGTTAEEAMSR	BCL2 associated athanogene 6	Q9Z1R2	2.923	0.04	upregulation
GTAGKS(ph)PDLSSQKR	phosphoinositide kinase, FYVE-type zinc finger containing	Q9Z1T6	20.127	0.00249	upregulation
RPMEEDGEEKSPS(ph)KK	interleukin enhancer binding factor 3	Q9Z1X4	5.932	0.00853	upregulation
HSGPNS(ph)ADSANDGFVR	heterogeneous nuclear ribonucleoprotein F	Q9Z2X1	5.534	0.0112	upregulation
KEQSDISIS(ph)PR	arginine/serine-rich coiled-coil 2	S4R1G6	169867.578	0.0000208	upregulation

Phosphopeptides regulated by insulin, but not affected by SSE. 3T3-L1 adipocytes at the 14th day of differentiation were treated with SSE (70 μg/ml) or insulin (100 nM) for 30 min. Phosphoproteomic analysis was performed as described in Materials and Methods. Only phosphopeptides that were significantly affected by insulin, but not affected by SSE are presented. P-value was calculated by Student's *t*-test, compared to control, untreated cells.
